# Chemotherapy reprograms miRNA expression profiles in apoptotic extracellular vesicles from medulloblastoma cells, regulating pro- and anti-proliferative effects on recipient drug-naïve cells

**DOI:** 10.1186/s12964-025-02241-9

**Published:** 2025-06-10

**Authors:** Rosa Mistica C. Ignacio, Helen Forgham, Zerong Ma, Anya Jensen, George Sharbeen, Juanfang Ruan, David S. Ziegler, Maria Tsoli, Phoebe A. Phillips, Chelsea Mayoh, Maria Kavallaris, Joshua McCarroll

**Affiliations:** 1 Children’s Cancer Institute, Lowy Cancer Research Centre, UNSW Sydney, Sydney, NSW Australia; 2 School of Clinical Medicine, UNSW Medicine & Health, UNSW Sydney, Sydney, NSW Australia; 3 University of New South Wales Centre for Childhood Cancer Research, UNSW Sydney, Sydney, NSW Australia; 4 Australian Centre for Nanomedicine, UNSW Sydney, Sydney, NSW Australia; 5 UNSW RNA Institute, UNSW Sydney, Sydney, NSW Australia; 6 Australian Institute for Bioengineering and Nanotechnology, The University of Queensland, Brisbane, QLD Australia; 7 Pancreatic Cancer Translational Research Group, Adult Cancer Program, Lowy Cancer Research Centre, UNSW Sydney, Sydney, NSW Australia; 8 School of Biomedical Sciences, Faculty of Medicine & Health, UNSW Sydney, Sydney, NSW Australia; 9 Electron Microscope Unit, Mark Wainwright Analytical Centre, UNSW Sydney, Sydney, NSW Australia; 10 Kids Cancer Centre, Sydney Children’s Hospital, Sydney, NSW Australia

**Keywords:** Medulloblastoma, Extracellular vesicles, Cisplatin, MiRNA

## Abstract

**Background:**

Extracellular vesicles (EVs) play a crucial role in intercellular communication. While the effects of EVs released from living or non-dying cancer cells are well characterized, the impact of EVs released from chemotherapy-treated or apoptotic cancer cells is less understood. This study investigated the effects of the chemotherapy agent cisplatin on EV release and miRNA content in apoptotic medulloblastoma cells, as well as their influence on the growth of drug-naïve recipient cancer cells.

**Methods:**

EVs were isolated from cisplatin-treated and untreated SHH and group 3 medulloblastoma cells, as well as from the blood of mice with orthotopic medulloblastoma tumors. EVs were characterized using nanoparticle tracking analysis, cryo-TEM, and western blotting, and their impact on the growth of recipient medulloblastoma cells in 2D and 3D cultures was assessed. EV-miRNAs were analyzed using small RNA sequencing and qPCR, and the effects of candidate miRNA overexpression on medulloblastoma cell growth and apoptosis were evaluated.

**Results:**

We demonstrate that apoptotic SHH and group 3 medulloblastoma cells secrete increased numbers of EVs (size range 150–600 nm) both in vitro and in vivo. EVs isolated from cisplatin-treated SHH and group 3 medulloblastoma cells were internalized by recipient medulloblastoma cells and exhibited distinct effects on their growth. EVs from cisplatin-treated SHH medulloblastoma cells reduced clonogenic growth in recipient drug-naïve medulloblastoma cells, whereas EVs from cisplatin-treated group 3 medulloblastoma cells enhanced the clonogenic and sphere-forming capacity of recipient cells. These contrasting effects were associated with significant alterations in EV-miRNA expression profiles between untreated and cisplatin-treated SHH and group 3 medulloblastoma cells. Notably, miR-449a was found to be upregulated in EVs from cisplatin-treated SHH medulloblastoma cells, and its overexpression in medulloblastoma cells led to potent inhibition of growth.

**Conclusions:**

Our findings demonstrate, for the first time, that cisplatin-treated medulloblastoma cells from distinct molecular subgroups secrete EVs with altered miRNA expression profiles that either inhibit or promote the growth of recipient cancer cells. This underscores the potential of targeting EV-mediated communication as a novel therapeutic strategy in medulloblastoma.

**Supplementary Information:**

The online version contains supplementary material available at 10.1186/s12964-025-02241-9.

## Background

Brain cancer is the leading cause of cancer-related death in children [1, 2]. Medulloblastoma accounts for approximately 25% of childhood brain tumors, making it the most common malignant brain tumor diagnosed in children [1, 2]. It is a neuroectodermal tumor of the cerebellum classified into four subgroups based on molecular differences, namely, WNT (Wingless signaling pathway activated), SHH (Sonic hedgehog signaling pathway activated), and non-WNT/non-SHH designated as group 3 and group 4 [1, 2]. Recently, these subgroups have been further divided into subtypes based on unique molecular alterations [3]. Treatment includes surgery, radiation, and chemotherapy, which together have produced varying survival outcomes for each of the four subgroups [1, 2]. WNT tumors have an excellent 5 year survival rate of over 90%, SHH and group 4 tumors have intermediate 5-year survival rates of approximately 70–80%, while group 3 tumors have a 5-year survival rate of approximately 45–60% [4, 5]. Children who present with poor prognostic factors such as metastatic disease or group 3 tumors with MYC amplification are classified as having high-risk disease [4, 5]. Sadly, the 5-year survival rate for this group of patients is approximately 60%, and specific subgroups have even worse outcomes. To change these dismal statistics, there is a need to increase our understanding of the mechanisms that regulate medulloblastoma cell growth and survival so that effective therapeutic strategies can be developed to combat this disease.

Extracellular vesicles (EVs) have received attention as major regulators of intercellular communication. EVs are nanosized lipid-bilayer vesicles containing biologically active proteins, lipids, RNA and DNA that are secreted by tumor and non-tumor cells [6]. Currently, there are three major classes of EVs which vary in size, biogenesis and cellular release: (1) exosomes (30–150 nm), which are formed inside multivesicular bodies and released through exocytosis, (2) microvesicles (100–1000 nm), which are shed directly from the plasma membrane; and (3) large apoptotic bodies (> 1 μm), which are produced during programmed cell death [7, 8]. Proteins typically found to be enriched in exosomes and microvesicles and are often used to confirm isolation of EVs in biological fluids including, tetraspanins, syntenin, Alix and Tumor Susceptibility gene 101 (TSG101), all of which are involved in EV biogenesis or cargo loading [7, 8]. EVs can be isolated in body fluids such as blood, urine, saliva, and cerebrospinal fluid, both in healthy and pathological conditions [7, 8]. Cancer cells use EVs to communicate with neighboring or distant tumor and non-tumor cells to transfer their biological contents, which in turn regulate gene expression and signaling pathways in recipient cells to promote growth or survival as well as metastases at distant sites [9]. For example, EVs released from metastatic breast cancer cells were demonstrated to alter the function of the major cell types that comprise the blood-brain barrier (BBB), which increased BBB permeability, allowing breast cancer cells to colonise into the brain [10].

Limited information exists on the role played by EVs in regulating medulloblastoma growth. A study by Epple *et al.* was the first to characterise EVs released by drug-naïve medulloblastoma cells [11]. Proteomic analysis on EVs isolated from SHH and group 3 medulloblastoma cells showed an abundance of proteins that had the capacity to impact the growth of EV-recipient cells [11]. Indeed, they demonstrated that EVs isolated from group 3 medulloblastoma cells could promote the growth of EV recipient medulloblastoma cells via activation of the mitogen-activated protein kinase/extracellular signal-regulated kinase (MAPK/ERK) signaling pathway [11]. Expanding on these findings, Zhu *et al. *reported that medulloblastoma cells belonging to the group 3 subgroup released EVs with sizes ranging from 30 to 150 nm which were internalized by recipient medulloblastoma cells [12]. The EVs induced the migratory and invasive capacity of the recipient cells [12]. These changes in cell function correlated with increased expression of several EV-miRNAs, which activated ERK/Ras signaling in the EV-recipient cells. Further support for a role of EVs in promoting aggressive medulloblastoma growth was recently demonstrated by Albert *et al.* where they showed that upregulated expression of an EV-related gene expression signature was an independent prognostic indicator for high-risk disease across all four molecular medulloblastoma subgroups [13].

Most studies to date have characterized the biological function and/or contents of EVs released from drug-naïve / non-dying cancer cells. However, there is a growing body of evidence that shows dying, or apoptotic cancer cells also release high numbers of EVs which collectively are known as apoptotic-extracellular vesicles (Apo-EVs) [13]. Three subtypes of Apo-EVs have been identified, with apoptotic bodies as described above being the first identified and most extensively characterized subtype [13]. Recent advancements in EV isolation and characterization techniques have led to the discovery of an additional two Apo-EV subtypes known as apoptotic multivesicles (100–1000 nm) and ApoExo’s (< 150 nm) [13]. Both subtypes often contain protein markers that are present in classical multivesicles and exosomes released from non-dying cells such as CD63, TSG-101, Alix and Flotillin, however, they also contain proteins typically not found in classical EVs such as phosphatidylserine, Annexin A1 and caspase 3 [14]. Interestingly, studies have shown that apoptotic bodies and Apo-EVs have different biological cargos and effects on recipient cells [14]. No studies have examined whether Apo-EVs released from medulloblastoma cells effect recipient cancer cell growth.

Here we report for the first time that apoptotic SHH and group 3 medulloblastoma cells exposed to a sublethal concentration of cisplatin, secrete large numbers of small and large Apo-EVs in vitro and in vivo. The Apo-EVs can be internalized by recipient drug-naïve medulloblastoma cells. Apo-EVs isolated from SHH medulloblastoma cells decreased clonogenic growth of recipient medulloblastoma cells. In contrast, Apo-EVs isolated from group 3 medulloblastoma cells increased clonogenic growth and spheroid forming capacity of the cells. The observed differences in recipient cell growth correlated with marked alterations in the miRNA expression profile of Apo-EVs isolated from SHH and group 3 medulloblastoma cells. Apo-EVs isolated from SHH medulloblastoma cells had increased expression of tumor suppressor miRNAs including, miR-449a compared with Apo-EVs isolated from group 3 medulloblastoma cells. Overexpression of miR-449a in SHH medulloblastoma cells led to potent anti-proliferative effects in the absence or presence of cisplatin.

## Methods

### Cell culture

SHH-DAOY cells were obtained from the Children’s Cancer Institute Cell Bank (New South Wales, Australia). SHH-UW228 cells were a kind gift from Professor Nick Gottardo and Dr Raelene Endersby from the Telethon Kids Institute, Perth Children’s Hospital, Perth, Western Australia. Group 3-D283 Med ATCC ^®^ HTB-185™ and group 3-D341 Med ATCC ^®^ HTB-187™ cells were purchased from The American Type Culture Collection (ATCC, VA, USA). The culturing of cell lines was performed as described by the manufacturer’s guidelines and were maintained in Dulbecco’s Modified Eagle Medium (DMEM), supplemented with 10% fetal bovine serum (FBS). The purity of all cell lines was established using short tandem-repeat profiling (SHH-DAOY: CellBank Australia; group 3-D283, group 3-D341: ATCC and SHH-UW228: Garvan Institute of Medical Research, Sydney, New South Wales, Australia). SHH-DAOY cells were transduced to constitutively express green fluorescence protein and luciferase in accordance with the manufacturer’s guidelines, using premade lentiviral particles expressing a fusion target of GFP-Luciferase / firefly (GenTarget, CA, USA) under the notifiable low risk dealing (NLRD 22 − 19). All cells were maintained in a humidified incubator at 37 °C and 5% CO_2_ and tested negative for Mycoplasma contamination every three months. All cell lines were negative for mycoplasma.

### Cisplatin treatment and measurement of cell viability and apoptosis

To determine a concentration of cisplatin which will result in 50% reduction in cell viability, SHH-DAOY and group 3-D283 cells were treated with increasing concentrations of cisplatin (10 µM, 30 µM, 60 µM) in a complete medium supplemented with 10% fetal bovine serum (FBS) for 3 h. After treatment, the media containing cisplatin was removed and cells were washed with Phosphate Buffered Saline (PBS) and replaced with fresh media. Forty-eight hours later, cells were harvested and the number of live-and dead cells counted using trypan blue staining as previously described [15].

To confirm that short-term exposure to 30 µM cisplatin induced apoptosis in SHH and group 3 medulloblastoma cells, SHH-DAOY and group 3-D283 cells were treated with 30 µM cisplatin as described above. Forty-eight hours post-treatment, cells were harvested, and apoptosis was measured using Annexin V-PE/7-AAD staining (BD Biosciences, CA, USA) according to the manufacturer’s instructions. Cells were analyzed using BD FACS Canto II flow cytometer and the distribution of cells in early and late apoptosis was quantified using FlowJo v10.8.1 (FlowJo, OR, USA) software. In a separate set of experiments, SHH-DAOY and group 3-D283 cells were treated with 30 µM cisplatin as described above. Whole cell lysates were harvested and phosphorylated-Histone H2AX (p-H2AX) a marker of DNA damage, cleaved-caspase 3 and cleaved-poly [ADP-ribose] polymerase (PARP) markers of apoptosis were measured by western blotting as described below.

### Isolation of extracellular vesicles from cisplatin treated and non-treated Medulloblastoma cells

To isolate large and small extracellular vesicles (EVs) from cisplatin-treated and non-treated medulloblastoma cells, SHH-DAOY, SHH-UW228, group 3-D283 and group 3-D341 cells were grown in T150 cm^2^ tissue culture flasks until they reached 70% confluency. At confluency, cell culture media was removed, and cells were washed with PBS and incubated with fresh DMEM cell culture media supplemented with 10% exosome depleted FBS that was produced by ultracentrifugation at 100,000 x *g* for 18 h at 4 °C as described previously [16]. Cells were treated with or without 30 µM cisplatin for 3 h. To limit the presence of cisplatin in the medium following treatment, the media containing cisplatin was removed and cells were washed with PBS and replaced with fresh complete media. Two hours later, the cells were washed again using PBS and replaced with fresh complete media containing 10% exosome depleted FBS. Forty-eight hours post-treatment, media was collected and centrifuged at 350 x *g* for *5* min. This was followed by additional centrifugation steps at 1,400 x *g* for 5 min, filtration through a 0.8 μm sterile filter to remove cell debris and another centrifugation at 4,000 x *g* for 10 min at 4 °C to remove large apoptotic bodies. EVs from cisplatin treated and non-treated cells were then pelleted using ultracentrifugation at 20,000 x *g* for 3 h at 4 °C using Optima XE-100 Ultracentrifuge (Beckman Coulter Australia Pty Ltd, New South Wales, Australia), and resuspended in 300 µL of sterile PBS along with an equal volume of total exosome isolation reagent (Thermo Fisher Scientific, MA, USA) in a fresh 1.5 mL Eppendorf tube. After an overnight incubation, the solution containing EVs was centrifuged at 17,000 x *g* for 1 h at 4 °C and the pellet containing EVs was washed with PBS. Finally, EVs were suspended with 50 µL of PBS or RIPA cell lysis buffer for physical and protein analysis. If required, EVs were stored at 4 °C and used within 1 week.

### Isolation of extracellular vesicles from mice with orthotopic Medulloblastoma tumors treated with or without cisplatin

All animal experiments were approved by the University of New South Wales Animal Care and Ethics Committee according to the Animal Research Act, 1985, under the ethics number ACEC 19/11B (New South Wales, Australia) and the Australian Code for the Care and Use of Animals for Scientific Purposes (2013). Orthotopic surgery to implant GFP/Luciferase expressing SHH-DAOY cells was performed on 7-week-old Balb/c nude mice with an average weight of 16–18 g, purchased from the Australian Resources Centre (Western Australia, Australia). Prior to surgery, mice were administered Temgesic™ (Provet Pty Ltd, New South Wales, Australia) subcutaneously for pain relief at a dose of 0.1 mg/kg. Mice were anesthetized using isoflurane delivered into an induction chamber at a flow rate of 1 L/min. The mice remained under anesthetic once positioned within the stereotaxic device (SDR Scientific, New South Wales, Australia). Using predefined co-ordinates for the site of medulloblastoma pathogenesis, a bore hole was made in the skull directly above the cerebellum [17]. Luciferase expressing SHH-DAOY cells (5 × 10^5^) were delivered using a 28G needle mounted on a Hamilton syringe, directly into the right side of the cerebellum and the incision was closed using a surgical glue. Two weeks post cell inoculation, mice were randomized into treatment groups based on bioluminescence and treated with cisplatin (1.5 mg/kg, i.p.) or saline (control) every other day for 12 days. Animals were weighed every second day, and bioluminescent imaging of tumor burden using the IVIS Lumina platform (Caliper Life Sciences, MA, USA) was performed mid-point as previously described [15, 18]. Forty-eight hours after the last cisplatin treatment, all mice were euthanized. Whole brains were collected and fixed with 4% paraformaldehyde for histological analysis. Blood was extracted by cardiac puncture using a 25G syringe and allowed to clot for 30 min at room temperature in an upright undisturbed position. To isolate intact EVs from whole blood, samples were centrifuged at 2,000 x *g* for 10 min at room temperature to separate serum. Pooled serum from both treatment groups (> 6 mice/group) were centrifuged at 3,000 x *g* for 15 min at 4 °C to remove cell debris. Serum samples were pooled and diluted 1:1 with PBS and passed through a 0.8 µM filter. EVs were isolated as described above for in vitro EV isolation [ultracentrifugation followed by addition with Total Exosome Isolation Reagent (from serum; Thermo Fisher Scientific) according to the manufacturer’s instructions. Pelleted EVs were suspended in PBS and subjected to nanoparticle tracking analysis and cryo-TEM as described below.

### Nanoparticle tracking analysis

EV size distribution and concentration / mL were measured using a Nanosight NS300 instrument (Malvern Panalytical LTD, New South Wales, Australia). EVs suspended in PBS (1:400 dilution) were loaded into the sample chamber before moving through the path of a laser beam and video captured as they passed through a 20X objective microscope lens. The size and concentration of EVs were quantitated using Malvern Nanoparticle Tracking Analysis software.

### Cryo-Transmission Electron microscopy

EVs isolated from cisplatin-treated medulloblastoma cells or whole blood of mice treated with or without cisplatin were absorbed onto glow discharged copper grids (Quantifoil R2/2, Quantifoil Micro Tools) and blotted for 5 s in a 100% humidity chamber at 4 °C, with a blotting force of 5. The grids were then plunge-frozen in liquid ethane using a Vitrobot Mark IV device (Thermo Fisher Scientific). The grids were imaged using a Talos Arctica cryo-TEM (Thermo Fisher Scientific), operating at 200 kV with the specimen held at liquid nitrogen temperatures. Images were captured using a Falcon 3EC direct detector camera in linear mode.

### Western blotting

Western blotting was performed using 20 µg of EV protein extracts or whole cell extracts isolated from cisplatin-treated and non-treated medulloblastoma cells. In brief, isolated EVs from cell culture media and whole cell extracts were resuspended in a lysis buffer (radio-immunoprecipitation assay) containing protease and phosphatase inhibitors (Roche Diagnostics Australia, New South Wales, Australia) on ice for 30 min. Equal amounts of protein lysates were separated on 10% or 12% SDS PAGE gels and transferred to nitrocellulose as previously described [15, 18]. Membranes were probed with the following primary antibodies at 1:1000 dilution unless specified with an overnight incubation at 4 °C. Alix (3A9), Flotillin-2, Calnexin, cleaved caspase 3, cleaved PARP, phosphorylated Histone-gamma-H2AX (γH2AX), and CDK6 were purchased from Cell Signaling Technology (MA, USA). Antibodies against TSG101 [EPR7130(B)], CD63 and glyceraldehyde-3-phosphate dehydrogenase (GAPDH) were purchased from Abcam (Cambridge, UK). Antibodies against cancer stem cell markers, such as BCRP/ABCG2 and CD133, were also purchased from Abcam. Blots were visualized and band intensity was quantitated using methods described previously [15, 18].

### Extracellular vesicle uptake into drug-naïve Medulloblastoma cells

SHH-DAOY cells (4.4 × 10^4^) were used as EV-recipient cells and plated into 6-well culture plates. Twenty-four hours later, EVs isolated from the media of SHH-DAOY and group 3-D283 cells were stained with 1 mM 4,4-Difluoro-1,3,5,7,8-Pentamethyl-4-Bora-3a,4a-Diaza-*s*-Indacene [(BODIPY™), Invitrogen, MA, USA] TR Ceramide fluorescent dye for 20 min at 37 °C and added onto recipient cells. Four hours later, cells were fixed in fresh 4% paraformaldehyde, permeabilized with 0.1% Triton X for 15 min, washed three times with PBS and stained with ActinGreen™ 488 ReadyProbes^®^ reagent as per manufacturer’s guidelines (Thermo Fisher Scientific). Imaging of EV cellular internalization was performed using the Leica DLS TCS SP8 confocal microscope (Leica, Wetzlar, Germany).

### Cell colony formation assay

SHH-DAOY and group 3-D283 medulloblastoma cells were treated with 30 µM cisplatin for 3 h as described above. Cells not treated with cisplatin served as control. Forty-eight hours post-cisplatin treatment, EVs were isolated from cell culture media from cisplatin-treated and non-treated cells as described above. SHH-DAOY and SHH-UW228 drug naïve cells were used as the EV-recipient cells and treated with equal amounts of intact EVs (50 µg/mL) isolated from either SHH-DAOY or group 3-D283 donor cells treated with or without cisplatin once a day for three days (to mimic large number of EVs released from drug-treated cells). Cells grown in culture media served as controls. All cells were cultured for an additional 7 days and allowed to form colonies. Cell colonies were gently washed twice with PBS, fixed and stained with 0.5% crystal violet containing methanol for 1 h at room temperature, and then washed thrice with Milli-Q water. Digital images of cell colonies were obtained using the ChemiDoc imaging system (Coomassie mode, BioRad, CA, USA) and cell colonies (> 50 cells) were counted manually.

### Spheroid formation assay

SHH-DAOY and group 3-D283 cells were treated with or without 30 µM cisplatin for 3 h as described above. Forty-eight hours post-treatment, EVs were isolated from both cell lines described above. SHH-DAOY cells acted as EV-recipient cells and were grown in their standard culture conditions, harvested, and dissociated into single cell suspensions. Cells (1 × 10^3^) were seeded in neurosphere culture medium [DMEM/F-12, 2% B-27 supplement (Thermo Fisher Scientific), 20 ng/mL EGF (Millennium Science, Victoria, Australia), and 20 ng/mL bFGF (Millennium Science)], made fresh daily in ultra-low attachment round-bottom 96-well plates (Corning, AZ, USA) in a volume of 150 µL/well. On the same day, cells were treated with EVs (50 µg/mL) isolated from SHH-DAOY or group 3-D283 donor cells treated with or without cisplatin once a day for three days. Twenty-four hours after the final treatment, 2.5% Matrigel (In Vitro Technologies, New South Wales, Australia) was added to the supernatant containing the medullospheres to promote tight spheroid formation. Brightfield images were taken and recorded over 7 days using the IncuCyte S3 (Essen BioScience Inc, MI, USA).

### miRNA isolation from extracellular vesicles secreted by cisplatin-treated and non-treated Medulloblastoma cells

RNAse A (20 ng/mL; Promega Australia, New South Wales, Australia) was added to a suspension of EVs isolated from cisplatin-treated and non-treated SHH-DAOY cells for 15 min at 37 °C prior to miRNA isolation to digest any contaminating co-isolated extravesicular RNAs. miRNAs were extracted from intact EVs using Norgen’s microRNA Purification Kit (Norgen, ON, Canada) as per the manufacturer’s instructions. miRNA yield, quality, and size were verified using an Agilent Bioanalyzer 2100 with Small RNA Lab-on-a-Chip (Agilent Technologies, CA, USA). The miRNA was stored at -80 °C before further analysis.

### Next generation sequencing of miRNA in extracellular vesicles isolated from cisplatin-treated and non-treated SHH Medulloblastoma cells

Purified EV-miRNA samples collected from SHH-DAOY cells treated with or without 30 µM cisplatin were analyzed using next generation sequencing at the Ramaciotti Centre for Genomics, UNSW. Briefly, EV-miRNA samples were made ready for library preparation using the QIASeq miRNA library prep kit (Qiagen, Hilden, Germany) with 8 bp single indexes, as per manufacturer’s guidelines. Library yields were checked using the Qubit dsDNA high sensitivity kit, while the library profiles were checked using the LabChip DNA high sensitivity reagent kit. Samples from cisplatin-treated and non-treated cells were pooled equimolar and sequenced on the Illumina NextSeq 500 using the MID output 150 cycle kit run at 1 × 75 bp (Illumina, CA, USA) to generate an output of up to 20 million reads/sample. The sequencing run was loaded at 20 pM with 1% PhiX spike-in.

### Quantitative real-time PCR (qPCR)

The expression levels of selected miRNAs identified through next generation sequencing were validated in freshly isolated EVs by qPCR. Briefly, 50–100 ng miRNA was reverse transcribed using miRCURY LNA RT kit (Qiagen) according to the manufacturer’s guidelines. qPCR was then performed using miRCURY LNA miRNA probe PCR assay kit (Qiagen) run on a Applied Biosystems^™^ QuantStudio 3 Real-Time PCR platform (Fisher Scientific). Primers were obtained from Qiagen: miR-449a (geneglobe ID: ZP00001121), miR-1275 (geneglobe ID: ZP00000181), miR-223-3p (geneglobe ID: ZP00000464), SNORD48 (reference gene, geneglobe ID: ZP00004677) and U6 snRNA (reference gene, geneglobe ID: ZP00030496). Concentrations were calculated based on ΔC_t_ and normalized to SNORD48 or U6 snRNA.

### miR-1275, miR-449a mimic transfection, apoptosis, cell cycle analysis and cell colony growth

*Transfection*: hsa-miR-449a mimic (miRVANA: Assay ID: MC11127), hsa-miR-1275 mimic (miRVANA: Assay ID: MC13445), and miRVANA^™^ miRNA mimic negative control (negative control miRNA mimic) were purchased from Invitrogen. SHH-DAOY cells were transfected using Lipofectamine 2000 (Thermo Fisher Scientific) according to the manufacturer’s protocol. miR-449a and miR-1275 mimics, and negative control miRNA mimic were used at a final concentration of 10 nM for all experiments unless otherwise indicated.

#### Apoptosis

To measure apoptosis, adherent and floating SHH-DAOY cells were collected 48 h post treatment with 10 nM hsa-miR-449a mimic or 10 nM negative control miRNA mimic and stained with Annexin V-PE/7-AAD kit (BD Biosciences) according to the manufacturer’s instructions. Cells were analyzed using BD FACS Canto II and the distribution of cells in early, middle and late apoptosis were quantified using FlowJo v10.8.1 (FlowJo) software.

#### Cell cycle analysis

SHH-DAOY cells were seeded in 6-well plates and transfected with 10 nM hsa-miR-449a mimic or 10 nM negative control miRNA mimic using lipofectamine 2000 and incubated for 72 h. Adherent and floating cells were fixed using 70% ethanol for 24 h at -30 °C and washed with ice-cold PBS. The cells were stained with DNA staining solution (50 µg/mL propidium iodide (Sigma-Aldrich, MI, USA), 2 µg/mL RNase (Promega Australia) and 0.6% Triton X-100) for 20 min at 4 °C. Stained cells were examined on a BD LSRFortessa™ SORP X-20 flow cytometer. Histograms were generated using FlowJo v10.8.1 (FlowJo), and cell cycle analysis was performed using the Watson Pragmatic model, which fits Gaussian curves to each cell cycle phase.

#### Cell colony growth

Experiments examining the effect of transient increased miR-449a expression on the clonogenic capacity of SHH-DAOY cells in the presence or absence of cisplatin were conducted as follows. SHH-DAOY cells were transfected with 10 nM of hsa-miR-449a mimic or negative control miRNA mimic using lipofectamine 2000 for 2 h. Two hours post transfection, SHH-DAOY cells were plated into 6-well plates and left to adhere overnight. Cells were treated with 50 nM and 100 nM of cisplatin for 72 h. Media was removed and replaced with fresh culture media. Cells were allowed to form colonies for 7 days post-cisplatin treatment. Cell colonies were stained and counted as described above.

### Statistical analysis

Statistical analysis was performed using GraphPad Prism 9 (GraphPad Software, CA, USA) using the indicated tests. Student’s t-test was used when comparing changes in means between two treatment groups, and ANOVA in conjunction with multiple comparison test was used when comparing three or more treatment groups. Values of *p* ≤ 0.05 were reported as significant. Data presented were expressed as means ± standard error of the mean (SEM) of ≥ 3 independent experiments, unless specified otherwise.

## Results

### Cisplatin induces the release of extracellular vesicles from dying Medulloblastoma cells in vitro and in vivo

**Fig. 1:**
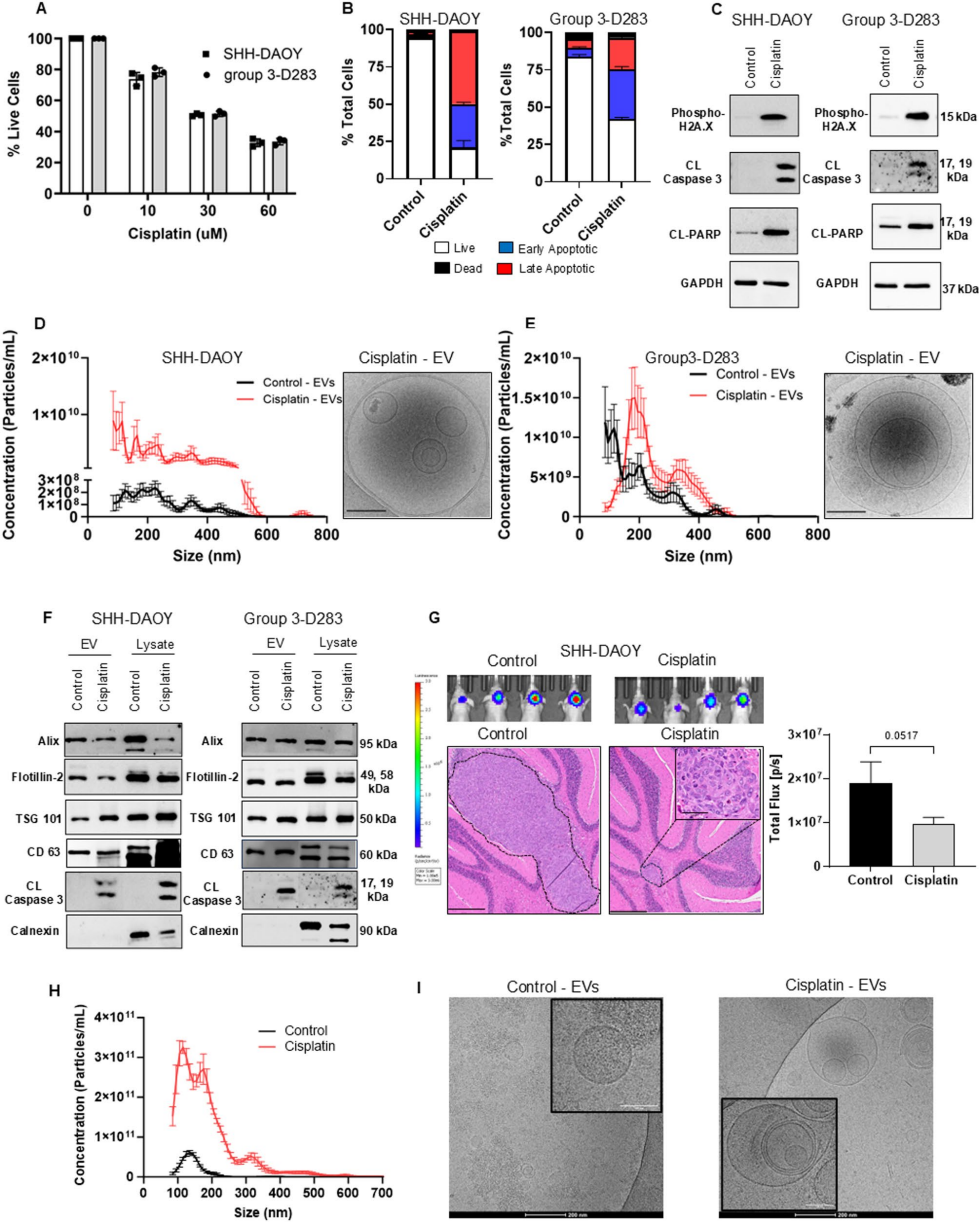
Cisplatin induces the release of extracellular vesicles from medulloblastoma cells in vitro and in vivo. **(A)** Graph showing cell viability of SHH-DAOY and group 3-D283 medulloblastoma cells treated with increasing concentrations of cisplatin. **(B)** Graphs showing Annexin V-PE/7-AAD positive SHH-DAOY and group 3-D283 cells treated with 30 µM cisplatin, non-treated cells served as controls. **(C)** Western blots of whole cell extracts showing 30 μM cisplatin induces DNA damage (phosphorylated-үH2A.X) and apoptosis (cleaved caspase-3 and cleaved PARP) in SHH-DAOY and group 3-D283 cells from three independent experiments. GAPDH was used as a protein loading control. **(D-E)** Nanoparticle Tracking Analysis and representative cryo-TEM images of extracellular vesicles released by medulloblastoma cells (SHH-DAOY and group 3-D283) treated with 30 µM cisplatin (red) compared to non-treated cells (black) (scale bar: 200 nM). **(F)** Western blot analysis of extracellular vesicle protein extracts or whole cell extracts collected from cisplatin treated and non-treated SHH-DAOY and group 3-D283 cells, *n* = 3 separate isolations. Data represent means ± SEM from three independent experiments. **(G)** Representative bioluminescent images of orthotopically-implanted Luciferase expressing SHH-DAOY cells in the brains of mice and hematoxylin and eosin stained tissue sections (scale bars: 400 µM and 50 µM for zoom image) showing a reduction in luciferase activity and tumor growth in mice treated with cisplatin compared to non-treated controls, > 6 mice/group. **(H)** Nanoparticle Tracking analysis showing increased numbers of extracellular vesicles released into the blood of mice treated with cisplatin (red line) compared to non-treated mice (black line), > 6 mice/group. **(I)** Cryo-TEM images showing that cisplatin induces the release of extracellular vesicles with different sizes into the blood compared to non-treated mice, > 6 mice/group (scale bars: 200 nM and 100 nM for zoom images)

The effects of exposure to a sub-lethal concentration of cisplatin on extracellular vesicle (EV) release were examined in medulloblastoma cells that belong to SHH (DAOY, UW228) or group 3 (D283, D341) molecular subgroups. First, we established the concentration of cisplatin that will result in approximately 50% cell death. SHH-DAOY and group 3-D283 cells were treated with increasing concentrations of cisplatin (10 µM, 30 µM and 60 µM) for 3 h after which the media containing cisplatin was removed and replaced with fresh culture media without the drug for 48 h. Cells incubated in media alone served as controls. As expected, 48 h post-cisplatin treatment a dose dependant decrease in the number of live cells was observed for both SHH-DAOY and group 3-D283 medulloblastoma cells treated with 10 µM, 30 µM and 60 µM cisplatin (Fig. 1A). At 30 µM, there was a 49 ± 0.50% and a 49 ± 0.98% decrease in the number of SHH-DAOY and group3-D283 cells, respectively (Fig. 1A). Annexin V-PE/7-AAD staining demonstrated that exposure to 30 µM cisplatin induced both early and late apoptosis in both cell lines (Fig. 1B). Western blotting confirmed the presence of DNA damage (γH2AX) which is the mode of action for cisplatin-induced cell death and cleaved caspase-3 and cleaved PARP both markers of apoptosis (Fig. 1C). Based on these findings short-term exposure to 30 µM cisplatin for both SHH and group 3 medulloblastoma cells was chosen for in vitro studies examining EV release and their biological function on recipient cells.

To examine EV release from SHH and group 3 medulloblastoma cells, we treated SHH-DAOY, SHH-UW228, group 3-D283, and group 3-D341 cells with 30 µM cisplatin to induce apoptosis. All cell lines treated with cisplatin secreted significantly higher numbers of EVs compared to drug-naïve (control) cells (Fig. 1D, E, Fig S1). Apoptotic SHH-DAOY cells secreted EVs with sizes ranging from 100 to 600 nm, with 85–115 nm being the most abundant (particle concentration of 10^9^/mL), compared to non-treated SHH-DAOY cells [particle concentration of 10^8^/mL, (Fig. 1D)]. Group 3-D283 cisplatin treated cells secreted significantly higher number of EVs compared to their drug-naïve control cells, with increased sizes ranging from 200 to 400 nm, with 205 nm being the most abundant [particle concentration of 10^10^/mL (Fig. 1E)]. SHH-UW228 and group3-D341 cells treated with cisplatin also secreted significantly more EVs compared to non-treated control cells (Fig. S1). Apoptotic SHH-UW228 cells secreted EVs with sizes ranging from 100 to 795 nm, with 165–215 nm being the most abundant (particle concentration of 10^10^/mL), compared to non-treated SHH-UW228 cells (particle concentration of 10^8^/mL) (Fig. S1). Group 3-D341 cisplatin treated cells secreted significantly higher number of EVs compared to their drug-naïve control cells, with increased sizes ranging from 195 to 500 nm, with 215 nm being the most abundant (particle concentration of 10^10^/mL) (Fig. S1). Cryo-TEM images of EVs isolated from cisplatin treated SHH-DAOY and group 3-D283 cells showed the presence of intact double layer membrane vesicles with varying sizes with smaller vesicles inside larger vesicles (Fig. 1D, E).

Proteins commonly found in exosomes and microvesicles, namely, CD63, Alix, Flotillin-2 and TSG101, were present in EVs isolated from both non-treated and cisplatin-treated SHH and group 3 medulloblastoma cells and their corresponding whole cell extracts (Fig. 1F). The absence of calnexin which resides in the lumen of the endoplasmic reticulum confirmed the absence of contaminating cellular debris in all EV samples, as calnexin was only expressed in whole cell extracts (Fig. 1F). Notably, there were some differences in expression of EV proteins between the non-treated and cisplatin treated cells as well as non-treated SHH and group 3 medulloblastoma cell lines. For instance, EVs isolated from cisplatin treated SHH-DAOY and group 3-D283 cells contained high levels of cleaved caspase-3, which is often found in EVs isolated from dying or apoptotic cells [19, 20] (Fig. 1F).

Next, we wanted to determine whether mice with orthotopic medulloblastoma tumors treated with cisplatin also secrete increased numbers of EVs. Mice with growing tumors were treated with cisplatin or drug vehicle. At endpoint, mice treated with cisplatin had a reduction in ex-vivo tumor bioluminescence, which correlated with a marked reduction in tumor size (Fig. 1G). Whole blood was collected and EVs were isolated and characterized from both treatment groups. Like the in vitro setting there was a pronounced increase in EV numbers in blood collected from cisplatin treated mice (Fig. 1H). Cisplatin treated mice secreted EVs with sizes ranging from 100 to 485 nm (particle concentration of 10^10^ − 10^11^) compared to non-treated (control) mice with EV sizes ranging from 100 to 200 nm (particle concentration of 10^9^ − 10^10^) (Fig. 1H). Cryo-TEM imaging confirmed the presence of intact EVs from both treatment groups (Fig. 1I). EVs isolated from whole blood of control mice had mostly circular shaped double membrane vesicles. While EVs isolated from blood of cisplatin treated mice contained both double and multilayer vesicles, with smaller vesicles inside larger ones (Fig. 1I). Collectively, these results demonstrate that exposure to cisplatin induces dying / apoptotic medulloblastoma cells to secrete large numbers of EVs with different sizes compared to non-treated cells.

### Extracellular vesicles isolated from cisplatin treated Medulloblastoma cells effect the growth of drug-naïve recipient cells

**Fig. 2:**
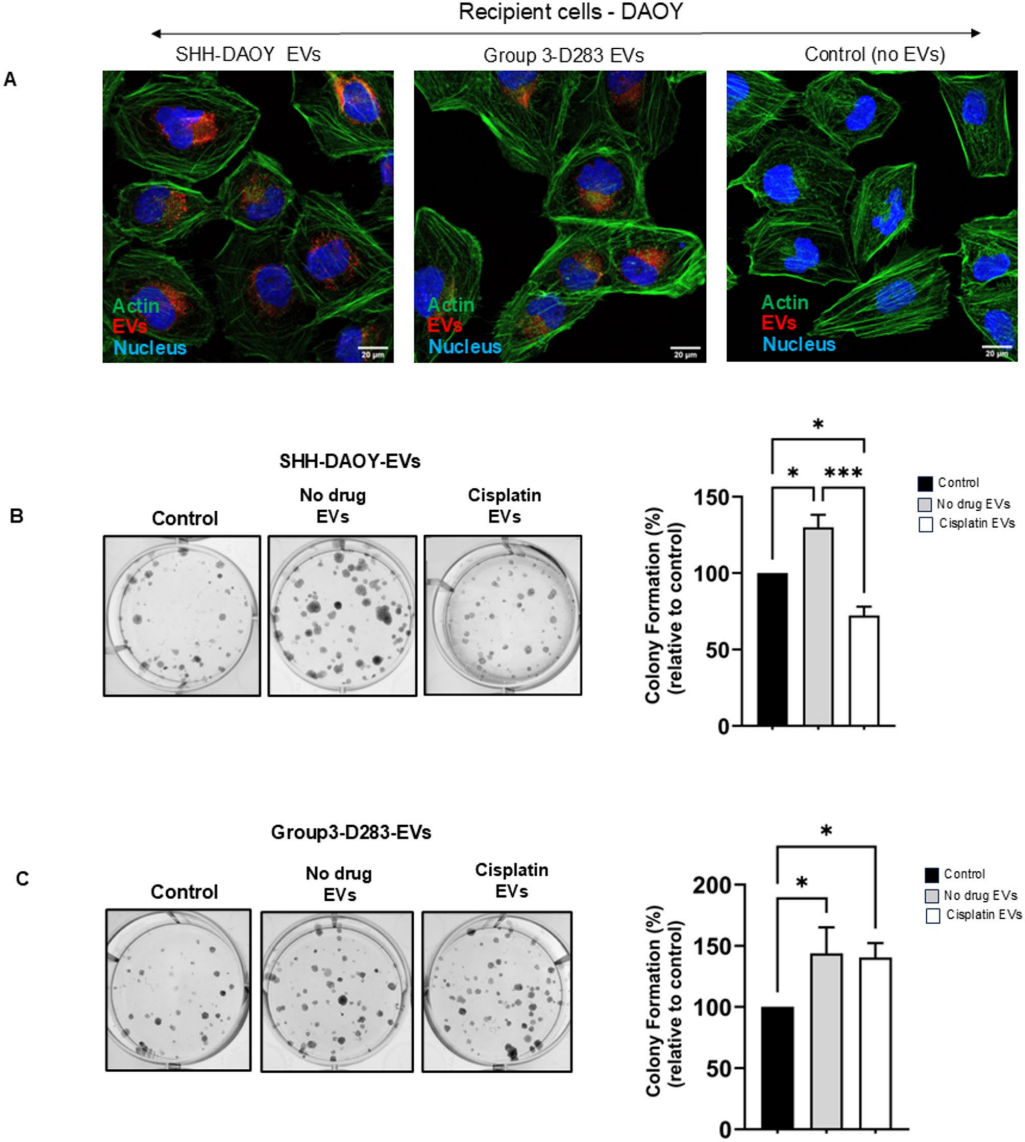
Extracellular vesicles from cisplatin-treated and non-treated medulloblastoma cells effect the clonogenic growth of drug-naïve cells. **(A)** Representative confocal microscope images showing that EVs (red) from SHH-DAOY and group 3-D283 cells (EV donor cells) are internalized by recipient SHH-DAOY cells; (Green = cell cytoskeleton, Blue = Nucleus, Red = EVs; scale bar: 20 µM). **(B)** Representative images of cell colonies and graphs showing the differences in clonogenic growth for recipient SHH-DAOY cells treated with EVs isolated from SHH-DAOY cisplatin-treated and non-treated cells. **(C)** Representative images of cell colonies and graphs showing differences in clonogenic growth for recipient SHH-DAOY cells treated with EVs isolated from group 3-D283 cisplatin-treated and non-treated cells. Recipient SHH-DAOY cells not treated with EVs served as controls. Data represent means ± SEM from three independent experiments, *p < 0.05, ***p < 0.001

To investigate whether EVs isolated from cisplatin treated medulloblastoma cells effected the growth of drug-naïve EV recipient medulloblastoma cells, SHH-DAOY and group 3-D283 cells were treated with or without cisplatin. EVs isolated from both cell lines (EV donor cells) were then added onto drug-naïve SHH-DAOY cells (EV recipient cells) (Fig. S2). SHH-DAOY cells grown in cell culture media (no EVs) served as controls (Fig. S2). To examine EV uptake into recipient cells, EVs from both donor cell lines (SHH-DAOY and group 3-D283) were labelled with BODIPY stain and added at equal amounts (50 µg/mL) onto recipient SHH-DAOY cells. EVs from both donor cells were rapidly internalized into the SHH-DAOY recipient cells in as little as 4 h, with strong accumulation around the cell nucleus (Fig. 2A). Next, EVs isolated from both donor cell lines treated with or without cisplatin were added onto recipient SHH-DAOY cells and clonogenic growth monitored over 7 days. EVs from both non-treated SHH-DAOY and group 3-D283 cells increased cell colony growth in recipient cells compared to control cells (no EV treatment) (Fig. 2B, C). However, EVs collected from the two donor cell lines (SHH-DAOY and group 3-D283) treated with cisplatin had opposing effects on the clonogenic growth of the recipient cells. EVs from cisplatin treated SHH-DAOY cells significantly decreased clonogenic growth in the recipient cells compared to cells treated with EVs collected from non-treated cells and controls (Fig. 2B). While EVs collected from cisplatin treated group 3-D283 cells increased clonogenic growth of recipient cells compared to control cells (Fig. 2C). The increase in growth was similar to that elicited by EVs collected from non-treated group 3-D283 cells (Fig. 2C). To validate these findings in an additional cell line, we added EVs isolated from donor SHH-DAOY cells treated with or without cisplatin onto recipient SHH-UW228 cells. The results were consistent with those observed in SHH-DAOY recipient cells: EVs from untreated DAOY cells increased cell colony growth in SHH-UW228 recipient cells compared to control cells (no EV treatment), while EVs from cisplatin-treated DAOY cells significantly decreased clonogenic growth in SHH-UW228 recipient cells compared to cells treated with EVs collected from non-treated cells and controls (Fig. S3).

**Fig. 3:**
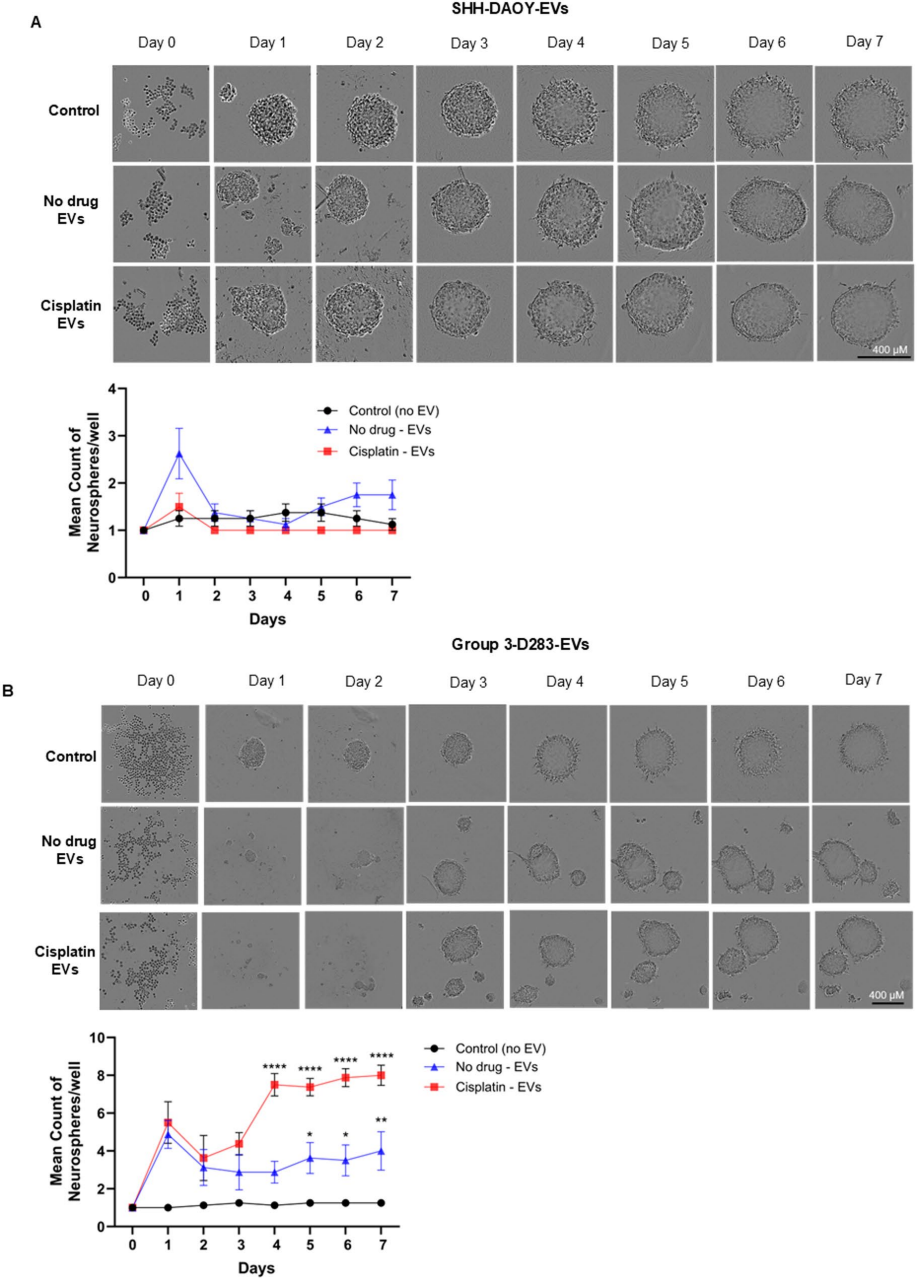
Extracellular vesicles isolated from cisplatin treated and non-treated medulloblastoma cells effect drug-naïve spheroid growth. **(A)** Representative images of SHH-DAOY spheroids and graph showing differences in spheroid growth for cells treated with EVs isolated from SHH-DAOY cisplatin-treated and non-treated cells. **(B)** Representative images of SHH-DAOY spheroids and graph showing differences in spheroid growth for cells treated with EVs isolated from group3-D283 cisplatin-treated and non-treated cells (scale bar: 400 µM). SHH-DAOY cells not treated with EVs served as controls. Data represent means ± SEM from three independent experiments, *p < 0.05, **p < 0.01, ****p < 0.0001

Next, we examined the effect of EVs on recipient spheroid forming capacity. Medulloblastoma cells when cultured under low adherence and allowed to form spheroids (medullosphere) have previously been reported to encourage the enrichment of cells with cancer stem cell properties [21]. Indeed, we showed that SHH-DAOY cells when grown as medullospheres in low adherence had increased expression of cancer stem cell markers, CD133 and ABCG2, when compared to the same cells grown in standard 2D culture (Fig. S4). Using this model, we added EVs isolated from donor SHH-DAOY and group 3-D283 cells treated with or without cisplatin onto recipient SHH-DAOY cells growing in low adherence. EVs collected from cisplatin treated and non-treated SHH-DAOY had no significant effect on recipient medullosphere growth after 7 days of culture compared to control cells (no EVs) (Fig. 3A). In contrast, EVs collected from non-treated group 3-D283 cells significantly increased the number of medullospheres compared to control cells (Fig. 3B). EVs isolated from cisplatin treated group3-D283 cells further increased the number of medullospheres compared to EVs collected from either the non-treated or control cells (Fig. 3B). Collectively, these findings suggest that medulloblastoma cells from SHH and group 3 molecular subgroups when exposed to cisplatin secrete large numbers of EVs which elicit positive-and-negative effects on the growth of recipient medulloblastoma cells.

### Cisplatin reprograms miRNA expression profile in extracellular vesicles secreted by Medulloblastoma cells

**Fig. 4:**
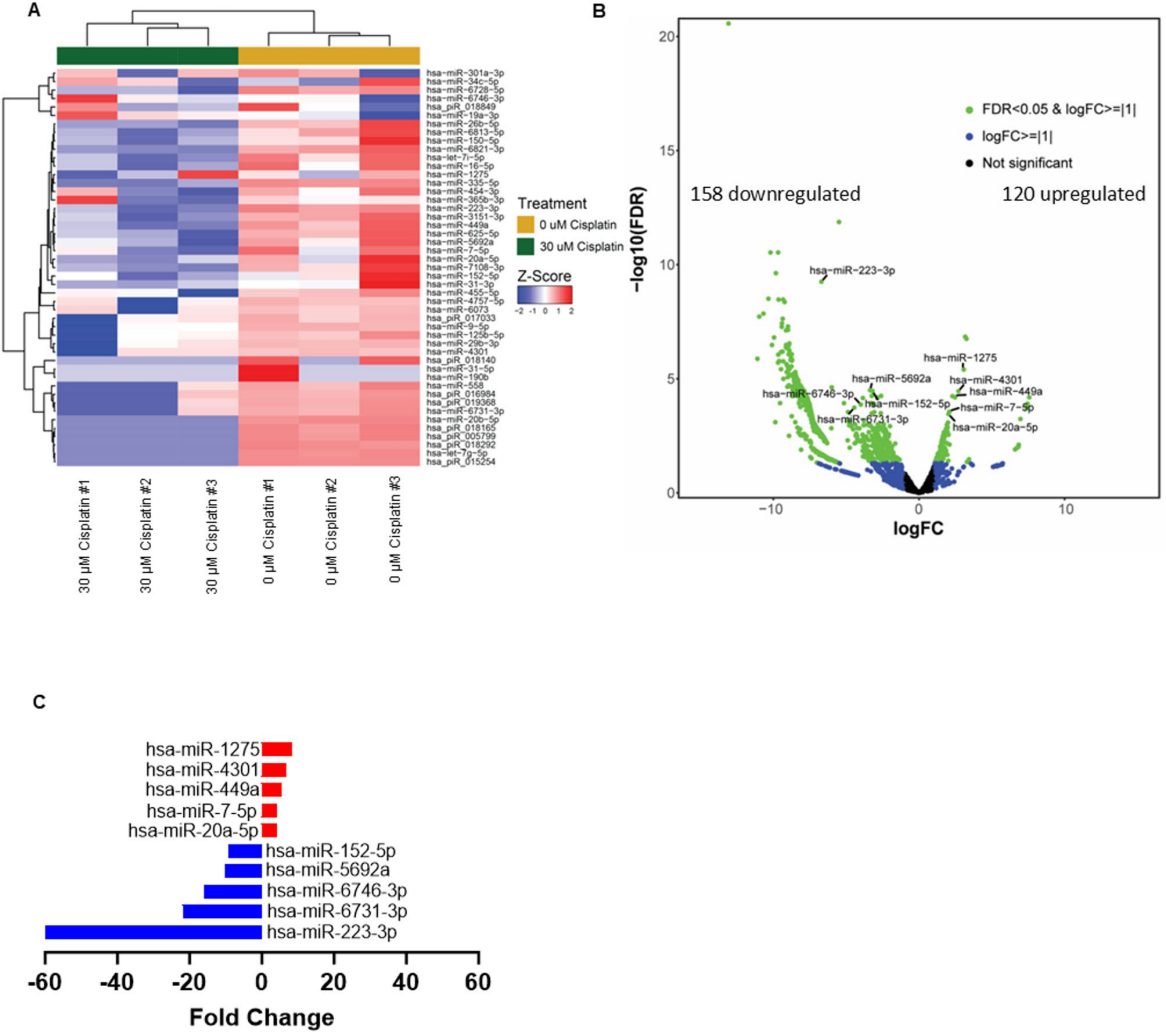
Cisplatin induces changes in the miRNA expression profile in extracellular vesicles secreted by medulloblastoma cells. **(A)** Heat map of differentially expressed miRNAs isolated from extracellular vesicles (EVs) secreted from SHH-DAOY cells treated with or without cisplatin. Data analyzed using small RNA-seq, *n* = 3 separate EV isolations. **(B)** Volcano plot of differentially expressed extracellular vesicle miRNAs isolated from cisplatin treated and non-treated SHH-DAOY cells. **(C)** Graph showing the top 5 up-and-downregulated miRNAs in extracellular vesicles isolated from cisplatin-treated SHH-DAOY cells compared to non-treated (control) cells

**Fig. 5:**
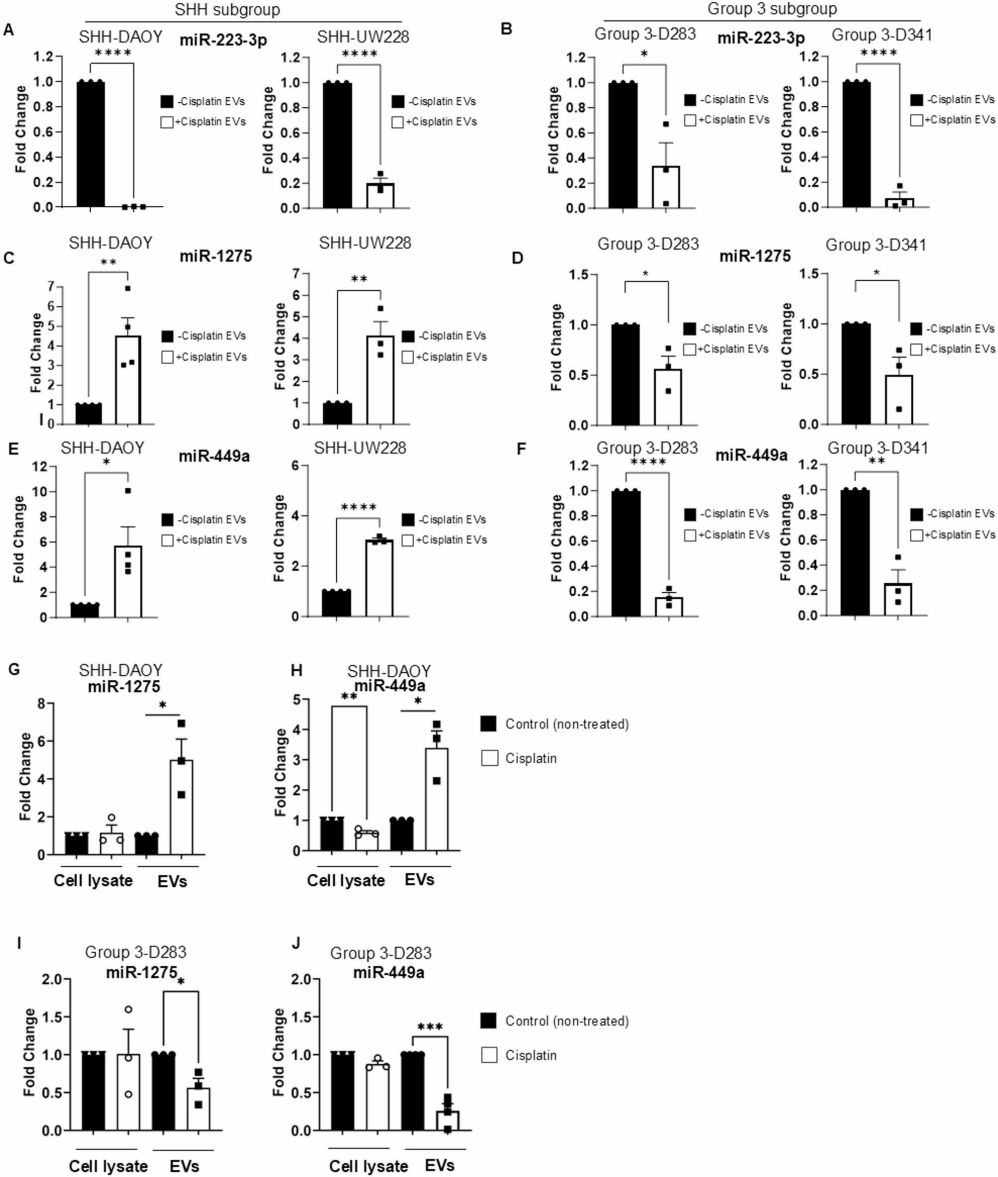
qPCR validated miRNA expression changes in extracellular vesicles from cisplatin-treated and non-treated medulloblastoma cells. **(A-F)** qPCR validation of several candidate miRNAs identified by small RNA-seq in extracellular vesicles isolated from cisplatin treated and non-treated SHH-DAOY, SHH-UW228, group 3-D283 and group 3-D341 cells;SNORD48 served as reference gene. **(G-H)** Measurement of miR-1275 and miR449a expression in whole cell extracts and isolated extracellular vesicles collected from cisplatin treated and non-treated SHH DAOY cells **(G-H)** and group 3-D283 cells **(I-J)**. SNORD48 served as reference gene. Data represent means ± SEM from three independent experiments, *p < 0.05, **p < 0.01, ***p < 0.001 ****p < 0.0001

To answer the question as to why EVs collected from cisplatin treated SHH and group 3 medulloblastoma cells have opposing effects on the growth of drug-naïve recipient cells, we performed small non-coding RNA-seq on EVs isolated from cisplatin treated SHH-DAOY cells, based on their inhibitory effect on clonogenic growth of recipient cells (Fig. 3A, Fig. S3). EVs isolated from non-treated SHH-DAOY cells served as a control. Using a 2.0-fold change, false discovery rate (FDR) ≤ 0.05 and a *p* value of < 0.05, we identified 120 upregulated and 158 downregulated miRNAs in EVs collected from cisplatin treated cells compared to EVs isolated from non-treated (control) cells (Fig. 4A, B). The top 5 upregulated EV-miRNAs, and top 5 downregulated EV-miRNAs, from cisplatin treated SHH-DAOY cells were identified (Fig. 4C). From these, we validated two upregulated miRNAs, miR-1275 and miR-449a and one downregulated miRNA, miR-223-3p using qPCR, and SNORD48 and U6 as the housekeeping reference genes in freshly isolated EVs collected from cisplatin treated-and non-treated SHH (DAOY and UW228) and group 3 (D283 and D341) medulloblastoma cells. miR-1275 and miR-449a have been identified as tumor suppressors in a number of different cancers [22–25], whereas miR-223-3p has been reported to exhibit both pro-tumor and anti-tumor effects depending on the cancer type [26, 27]. miR-223-3p was significantly lower in EVs collected from all four cell lines treated with cisplatin compared to non-treated controls (Fig. 5A, B, Fig. S5). While qPCR analysis confirmed that both miR-1275 and miR-449a were upregulated in freshly isolated EVs collected from cisplatin treated SHH-DAOY and SHH-UW228 cells compared to control EVs (Fig. 5C-E, Fig. S5). However, the opposite was observed in two different group 3 medulloblastoma cell lines (D283, D341) treated with cisplatin with miR-1275 and miR-449a expression significantly lower in isolated EVs compared to EVs collected from non-treated cells (Fig. 5D-F, Fig. S5).

Given the pronounced differences in miR-449a and miR-1275 expression in EVs collected from cisplatin-treated SHH and group 3 medulloblastoma cells, we examined whether these changes also occurred intracellularly. Intracellular levels of miR-1275 did not change in SHH-DAOY cells when treated with cisplatin compared to non-treated cells, with increased miR-1275 expression solely observed in the isolated EVs (Fig. 5G). miR-449a intracellular expression was slightly decreased in SHH-DAOY cells treated with cisplatin but upregulated by more than 3-fold in isolated EVs (Fig. 5H). Intracellular levels of both miR-1275 and miR-449a were slightly decreased in group 3-D283 cells treated with cisplatin, with the decrease in expression for both miRNAs more pronounced in the isolated EVs (Fig. 5I, J). These results suggest that cisplatin induces changes in the loading of both miR-1275 and miR-449a into EVs for both SHH and group 3 medulloblastoma cells.

### miR-449a inhibits Medulloblastoma clonogenic growth in the absence and presence of cisplatin

**Fig. 6:**
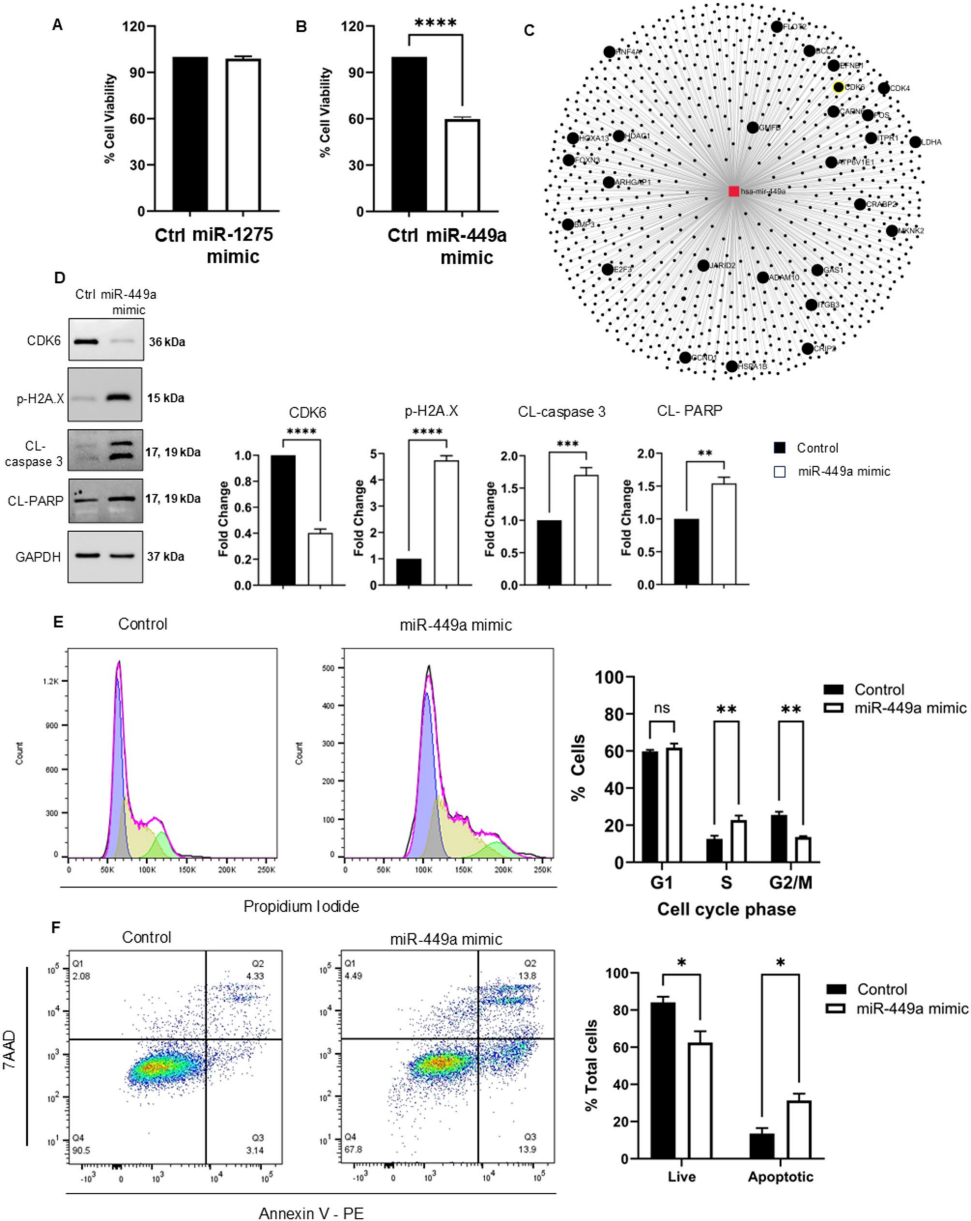
Overexpression of miR-449a inhibits SHH medulloblastoma cell growth and induces apoptosis. Cell viability graphs of SHH-DAOY cells transfected with **(A)** miR-1275 and **(B)** miR-449a mimics or non-functional miRNA / negative control miRNA mimic (control). **(C)** Graph showing predicted gene targets for miR-449a. **(D)** Western blot analysis showing decreased protein expression of miR-449a target CDK6 as well as increased DNA damage (phosphorylated үH2A.X) and increased cleaved caspase-3 and PARP (markers of apoptosis) in SHH-DAOY cells treated with miR-449a mimic. Cells treated with non-functional miRNA served as control. GAPDH was used a loading control. **(E)** Cell cycle analysis of SHH-DAOY cells transfected with miR-449a mimic or non-functional control miRNA mimic. **(F)** Graph showing Annexin V-PE/7-AAD staining of SHH-DAOY cells transfected with miR-449a mimic or non-functional miRNA mimic (control). Data represent means ± SEM from three independent experiments, *p < 0.05, **p < 0.01, ***p < 0.001, ****p < 0.0001

Given both miR-1275 and miR-449a were significantly upregulated in EVs isolated from cisplatin treated SHH-DAOY cells, and that these same EVs inhibited clonogenic growth of recipient cells, we performed miRNA overexpression experiments using miRNA mimics to determine their effect on medulloblastoma cell growth. First, we treated SHH-DAOY cells with miR-1275 or miR-449a mimics or non-functional miRNA (control). Increased miR-1275 expression in SHH-DAOY cells had no effect on SHH-DAOY cell viability 72 h post-treatment (Fig. 6A, Fig. S6). In contrast, increased expression of miR-449a led to a 40 ± 1.34% decrease in cell viability (Fig. 6B). Based on these findings, we focused on miR-449a and its role in regulating medulloblastoma growth. To understand how miR-449a reduces medulloblastoma cell growth, we identified potential miR-449a gene targets using several online target prediction tools including miRNet, TargetScan and miRDB (Fig. 6C). CDK6 was identified as one of several putative target genes for miR-449a (Fig. 6C). Therefore, given the functional importance of CDK6 in regulating the growth of SHH-medulloblastoma cells [28], we transfected SHH-DAOY cells with miR-449a mimic or non-functional (negative control miRNA mimic) miRNAs, and measured CDK6 protein expression 72 h post-treatment. Increased miR-449a expression in SHH-DAOY cells led to a pronounced reduction in CDK6 protein expression (Fig. 6D). This correlated with cell cycle arrest and a significant increase in the number of cells in S phase and decrease number of cells in the G2/M phase of the cell cycle (Fig. 6E). In addition, SHH-DAOY cells treated with miR-449a mimic had an increased number of apoptotic cells (Fig. 6F and D).

**Fig. 7:**
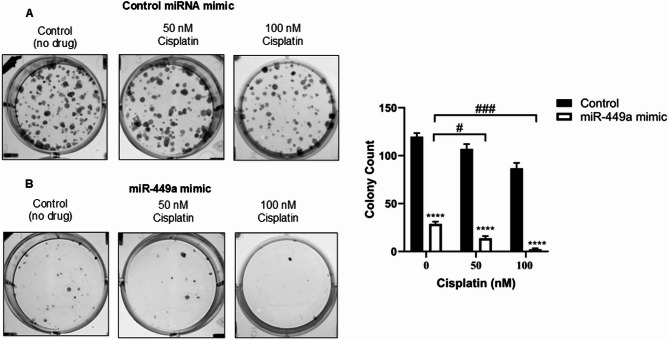
Overexpression of miR-449a inhibits SHH medulloblastoma colony growth in the absence or presence of cisplatin. Representative clonogenic cell growth images and graphs showing the differences in clonogenic growth of SHH-DAOY cells pre-treated with non-functional miRNA (control) **(A)** or miR-449a mimic **(B)** followed by treatment with cisplatin (50 nM and 100 nM). Data represent means ± SEM from three independent experiments, the asterisk (*) represents the p-value of the statistical test between non-functional miRNA (control) versus miR-449a mimic groups, ^#^*p* < 0.05, ^###^*p* < 0.001, ****p < 0.0001

Finally, to examine whether increased expression of miR-449a could inhibit cell colony growth in the presence or absence of cisplatin, SHH-DAOY cells were treated with either miR-449a mimic or non-functional miRNA (negative control miRNA mimic), with or without two sub-optimal concentrations of cisplatin. Cells treated with the miR-449a mimic formed significantly fewer cell colonies in the absence of cisplatin compared to those treated with the negative control miRNA mimic (Fig. 7A-B). The reduction in colony numbers was even more pronounced in the presence of cisplatin (Fig. 7A-B).

## Discussion

A wealth of information exists on how extracellular vesicles (EVs) secreted from non-dying cancer cells regulate tumor growth in a host of different cancers [6, 29]. However, there is limited knowledge on the role played by apoptotic-EVs (Apo-EVs) released from dying or apoptotic cells. In this study, we show for the first time that dying/apoptotic medulloblastoma cells from SHH and group 3 molecular subgroups after exposure to cisplatin secrete high numbers of small and large Apo-EVs. The Apo-EVs contained proteins commonly expressed in exosomes and multivesicles secreted by non-apoptotic cells, however they also expressed high levels of the apoptotic marker cleaved caspase-3, which was absent in EVs isolated from non-dying SHH and group 3-medulloblastoma cells. These findings are corroborated by other studies which have shown that cancer cells undergoing apoptosis secrete Apo-EVs. These Apo-EVs not only expressed protein markers common to EVs and multivesicles from non-dying cells, but also inherit apoptotic footprints from their parental cells, including high levels of caspases, phosphatidylserine, and/or the extrinsic apoptotic inducer Fas/FasL [30]. Several studies have suggested that caspase-3 activation plays an important role in regulating Apo-EV biogenesis. For example, Boing *et al.* showed that breast cancer cells which lack caspase-3 secrete low numbers of EVs [31]. However, when the cells were genetically engineered to express caspase-3 the number of secreted EVs increased by up to 5 fold compared to control (non-transfected) cells [31]. Moreover, the EVs contained high amounts of active capase-3 [31]. Interestingly, EVs containing active caspase-3 when internalized by recipient cells did not induce apoptosis [31]. The authors suggested that the dying cells load capase-3 into EVs for secretion as a means of regulating cellular homeostasis and limiting apoptotic death signals within the cells.

We observed contrasting effects of Apo-EVs collected from cisplatin-treated and dying SHH and group 3 medulloblastoma cells on the growth of Apo-EV-recipient medulloblastoma cells. Apo-EVs collected from dying SHH medulloblastoma cells inhibited the clonogenic growth of recipient cells. The same Apo-EVs had no obvious effect on spheroid numbers for recipient cells grown in 3D culture. In contrast, Apo-EVs collected from group 3 medulloblastoma cells increased both clonogenic growth and spheroid formation of recipient cells. The increase in spheroid numbers with Apo-EVs was two fold greater compared to EVs isolated from the same non-treated (living) cell line. Our data is consistent with other studies that have demonstrated that Apo-EVs secreted from different cell types modulate pro-or anti-tumor effects. He *et al. *reported that human lung cancer cells exposed to cisplatin or staurosporine and undergoing apoptosis, secreted large numbers of Apo-EVs [32]. These Apo-EVs were internalized by drug-naiive lung cancer cells and promoted metastases and stemness via activation of NF-κB signaling pathway which correlated with aggressive lung tumor growth in mice [32]. Wang *et al.* showed that Apo-EVs collected from mesenchymal stem cells when internalized by multiple myeloma cells induced extrinsic apoptosis which led to decreased tumor growth [33]. Further analysis of the secreted Apo-EVs showed they contained high levels of FasL, and directly interacted with multiple myeloma cells, thereby activating the FasL/Fas extrinsic apoptotic pathway [33].

EVs are a known source of miRNAs, and these miRNAs can be transferred to recipient cells to regulate their function [34]. There are reports in other cancers showing that exposure to chemotherapy or stress can alter the EV-miRNA expression profile [35]. We demonstrated that SHH medulloblastoma cells treated with cisplatin secrete Apo-EVs with a pronounced difference in miRNA expression profile compared to EVs collected from non-treated live cells. Of note, several of the differently expressed miRNAs such as miR-223-3p, miR-1275, miR-4301, miR-449a, miR-7-5p, and miR-20a-5p in Apo-EVs from the cisplatin-treated cells have been reported to act as powerful regulators of cell proliferation and survival in different cancer and non-cancer cells [22–25, 36–38]. We showed that miR-223-3p was downregulated in Apo-EVs isolated from cisplatin treated SHH and group 3 medulloblastoma cells compared to EVs collected from non-treated live cells. Low levels of miR-223-3p have been demonstrated in adult glioblastoma (GBM) tissue and increasing its expression using miR-223-3p mimics in GBM cells inhibited cell proliferation and migration by reducing the expression of several major inflammation-associated cytokines [26]. miR-223-3p levels in serum or isolated EVs have also been reported to have diagnostic value for different cancers. For instance, Papanota *et al*. reported that low levels of miR-223-3p in CD138 positive cells isolated from multiple myeloma patients correlated to inferior overall survival [39]. While Mukulski *et al.* demonstrated that high miR-223-3p serum levels in multiple myeloma patients served as an independent indicator for a complete response and longer overall survival following autologous hematopoietic stem cell transplantation [40]. No studies have examined the role of miR-223-3p in medulloblastoma cells. Therefore, future studies will be needed to ascertain its role in medulloblastoma cells and why exposure to cisplatin would decrease its levels in Apo-EVs.

Both miR-1275 and miR-449a were identified as upregulated in Apo-EVs collected from cisplatin-treated SHH medulloblastoma cells. Interestingly, the opposite trend was observed in Apo-EVs isolated from cisplatin-treated group 3 medulloblastoma cells. These differences in Apo-EV-miRNA expression between SHH and group 3 medulloblastoma cells were associated with pronounced differences in their effects on the growth of recipient cells, as described above. Therefore, we asked the question as to whether miR-1275 or miR-449a may be responsible for the observed decrease in clonogenic growth of recipient cells when treated with Apo-EVs isolated from cisplatin-treated SHH medulloblastoma cells given both miRNAs have reported tumor suppressor activity in different cancers. Transient overexpression of miR-1275 in SHH medulloblastoma cells had no effect on SHH medulloblastoma cell proliferation. In contrast, transient overexpression of miR-449a in SHH medulloblastoma cells significantly decreased cell proliferation. The tumor suppressive functions of miR-449a have been documented in different cancers [25, 41, 42]. Several studies have identified miR-449a as a key regulator of CDK6, which plays a critical role in modulating the cell cycle [43–45]. Loss of this miRNA leads to overexpression of CDK6 in cancer cells which promotes uncontrolled cell proliferation [46]. Our results confirmed previous findings in other cancer cell types that CDK6 is a target of miR-449a [45]. Treating SHH medulloblastoma cells with a miR-449a mimic led to a marked decrease in CDK6 protein expression. This correlated with reduced cell growth, cell cycle arrest, DNA damage and apoptosis. Moreover, we showed a reduction in cell colony growth in the presence or absence of cisplatin after overexpression of miR-449a in SHH medulloblastoma cells. Li *et al.* reported that a large proportion of medulloblastoma tumors have low miR-449a expression compared to normal cerebellum, with the exception of tumors belonging to the less aggressive WNT molecular subgroup which had high miR-449a expression [47]. Moroever, they showed that miR-449a expression was regulated by DNA hypermethylation in medulloblastoma cells and that treatment with a demethylation agent could increase miR-449a levels [47]. Therefore, it is possible that cisplatin may in part regulate its anti-cancer effect on SHH medulloblastoma cells via secretion of Apo-EV-miR-449a which when taken up by recipient cells increases miR-449a levels which leads to increased apoptosis and decreased cell proliferation. Future studies are required to explore the therapeutic potential of increasing miR-449a levels in medulloblastoma cells that belong to other subgroups either through the use of nanotechnology and delivery of miR-449a mimics or via treatment with DNA methylation inhibitors to normalize miR-449a overexpression to promote its tumor suppressor activities.

This study raises several questions that require attention in the future to expand our understanding of the role played by EVs and Apo-EVs in medulloblastoma. First, why do SHH and group 3 medulloblastoma cells treated with the same concentration of cisplatin and undergoing apoptosis have markedly different miRNA cargo in Apo-EVs secreted by the cells with these changes correlating with differential effects on the growth of drug naiive recipient cells. The most obvious difference between the two molecular subgroups is that group 3 cells often harbor amplification or overexpression of c-MYC or MYCN. Indeed, group 3 cell lines used in this study expressed high levels of c-MYC. Interestingly, a recent study by Kilinc* et al.* demonstrated that oncogenes such as MYC regulate the biogenesis and release of EVs with altererd miRNA expression profile [48]. Therefore, it is possible that the changes we observed in group 3 cell lines secreting high EV numbers and miRNA content in the presence or absence of cisplatin could be due to c-MYC amplication. However, further research will be needed to validate this hypothesis. We also showed that both SHH and group 3 medulloblastoma cells secrete a heterogeneous population of small and large EVs and Apo-EVs in the presence or absence of cisplatin. However, which subgroup of EVs or Apo-EVs is responsible for regulating the growth of recipient cells is not known. Studies that identify which subtype of EVs and Apo-EVs secreted by dying and/or live medulloblastoma cells effect the growth of recipient cells may help refine future therapeutic strategies that target their biogenesis and / or release. Finally, studies which examine changes in EV or Apo-EV miRNA cargo secreted by medulloblastoma cells in blood or CSF before, during or after treatment may serve as potential novel biomarkers to help clinicians determine whether a patient is responding to treatment or undergoing relapse.

Taken together, this study demonstrated that SHH and group 3 medulloblastoma cells when exposed to cisplatin secrete high numbers of Apo-EVs which have an altered miRNA expression profile. The Apo-EVs are internalized by recipient drug-naïve medulloblastoma cells and have anti-and pro-cancer cell growth effects. These findings suggest that Apo-EVs have an important role to play in regulating the effects of cisplatin and have the potential to identify new therapeutic strategies to increase the effectiveness of chemotherapy for medulloblastoma.

## Electronic supplementary material

Below is the link to the electronic supplementary material.

Supplementary Material 1: **Supplementary Fig. 1(A-B)** Nanoparticle tracking analysis graphs showing the number and size of extracellular vesicles released by SHH-UW228 and group 3-D341 cells treated with or without cisplatin. Data represent means ± SEM from three independent experiments.

Supplementary Material 2: **Supplementary Fig. 2**. Schematic for the experimental outline of using SHH-DAOY and group 3-D283 medulloblastoma cells as extracellular vesicle (EV)-donors and drug-naïve SHH-DAOY cells as EV-recipients to measure the effects of EVs on clonogenic and spheroid growth in 2D and 3D cultures.

Supplementary Material 3: **Supplementary Fig. 3**. Representative images of cell colonies and graphs showing the differences in clonogenic growth for recipient SHH-UW228 cells treated with EVs isolated from SHH-DAOY cisplatin-treated and non-treated cells. Recipient SHH-UW228 cells not treated with EVs served as controls. Data represent means ± SEM from three independent experiments, *p < 0.05, **p < 0.01.

Supplementary Material 4: **Supplementary Fig. 4**. Western blots showing protein expression of stem cell markers CD133 and ABCG2 after spheroid (medullosphere) formation. GAPDH was used as a protein loading control.

Supplementary Material 5: **Supplementary Fig. 5**: qPCR validation of candidate miRNAs, **(A-B)** miR-223-3p and **(C-D)** miR-449a in extracellular vesicles secreted by SHH-DAOY, SHH-UW228, group 3-D283 and group 3-D341 cells treated with or without cisplatin. U6 served as the reference gene. Extracellular vesicles isolated from non-treated cells served as control. Data represent means ± SEM from three independent experiments, *p < 0.05, **p < 0.01, ***p < 0.001****p < 0.0001.

Supplementary Material 6: **Supplementary Fig. 6**: Cell viability graph of SHH-DAOY cells transfected with increasing concentrations of miR-1275 mimic or non-functional miRNA (control) showing no effect on cell viability. Data represent means ± SEM from three independent experiments.

## Data Availability

No datasets were generated or analysed during the current study.

## Abbreviations

EVs: extracellular vesicles
WNT: Wingless signaling
SHH: Sonic hedgehog
TSG101: Tumor Susceptibility gene 101
MAPK/ERK: mitogen-activated protein kinase/extracellular signal-regulated kinase
Apo-EVs: apoptotic extracellular vesicles
DMEM: Dulbecco’s Modified Eagle Medium
FBS: fetal bovine serum
NLRD: notifiable low risk dealing
PBS: Phosphate Buffered Saline
үH2AX: phosphorylated Histone-gamma-H2AX
GAPDH: glyceraldehyde-3-phosphate dehydrogenase
BODIPY: 4,4-Difluoro-1,3,5,7,8-Pentamethyl-4-Bora-3a,4a-Diaza-s-Indacene
SEM: standard error of the mean
FDR: false discovery rate
GBM: glioblastoma
