# Supplementary figures and images for: Chemotherapy reprograms miRNA expression profiles in apoptotic extracellular vesicles from medulloblastoma cells, regulating pro- and anti-proliferative effects on recipient drug-naïve cells
Source: Cell Commun Signal. 2025 Jun 10;23:273. doi: 10.1186/s12964-025-02241-9 (PMC12150529; doi:10.1186/s12964-025-02241-9)

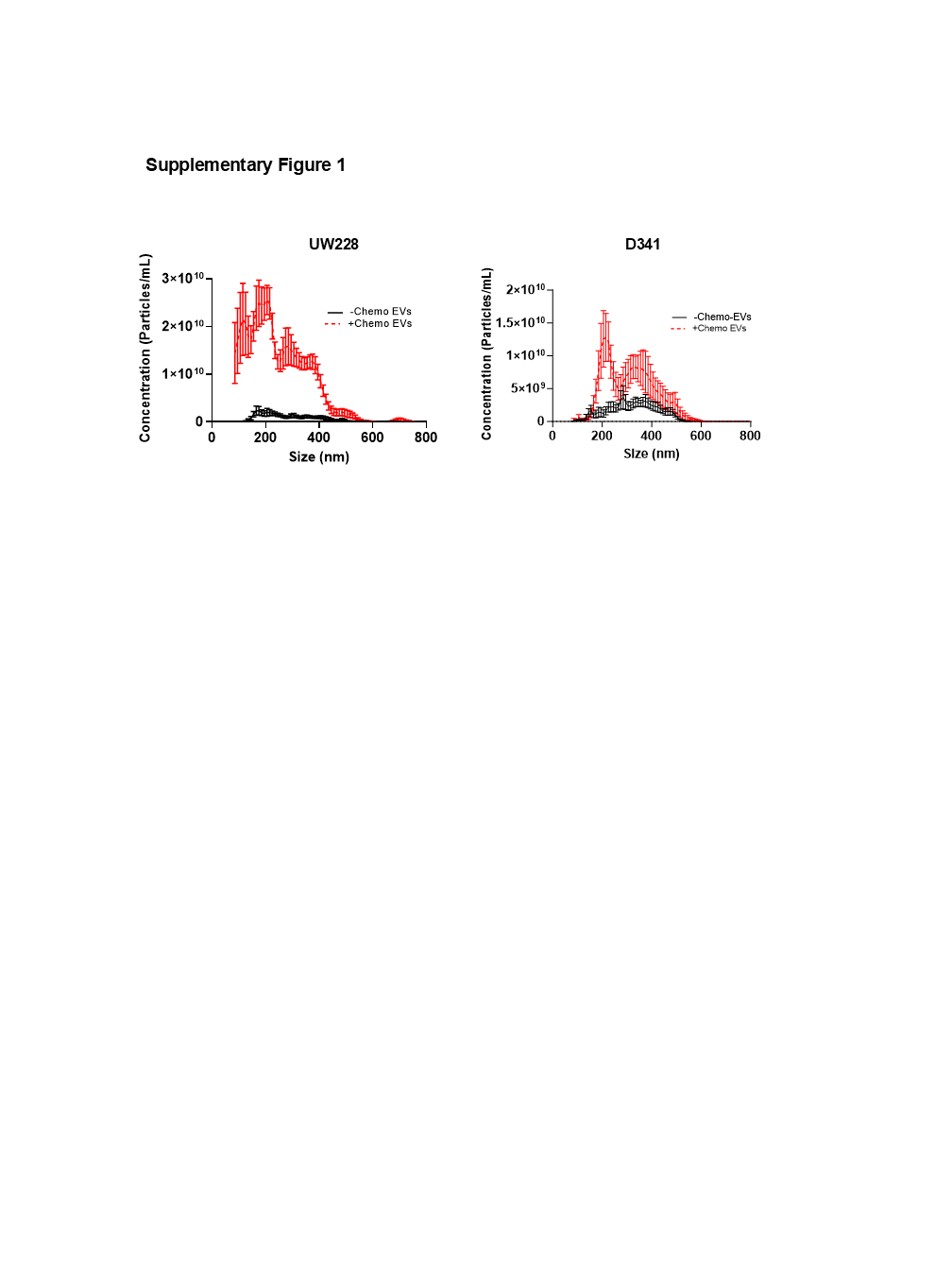

Supplement: Supplementary file 1 — Supplementary Material 1: Supplementary Fig. 1(A-B) Nanoparticle tracking analysis graphs showing the number and size of extracellular vesicles released by SHH-UW228 and group 3-D341 cells treated with or without cisplatin. Data represent means ± SEM from three independent experiments. [file 12964_2025_2241_MOESM1_ESM.tif]

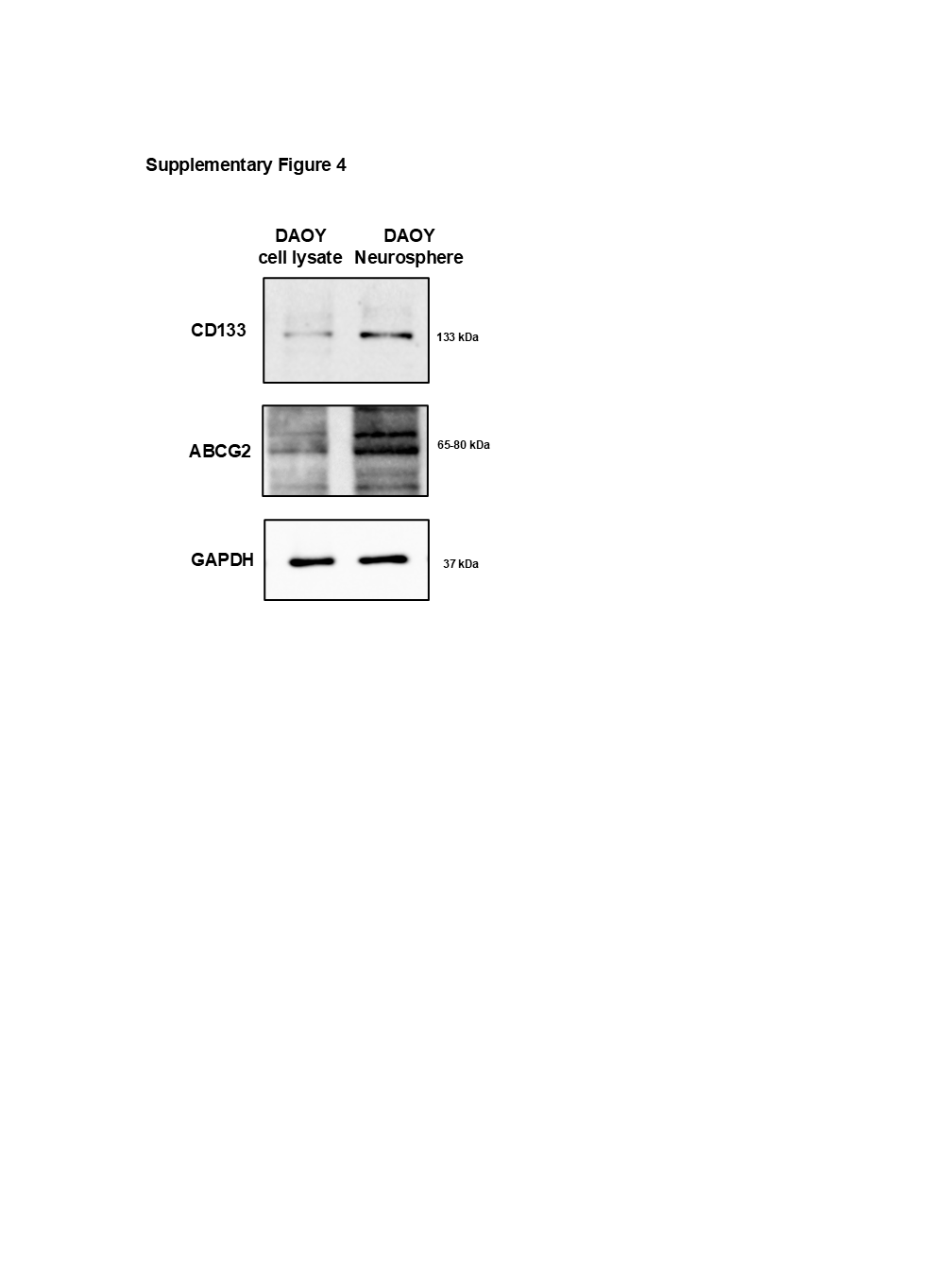

Supplement: Supplementary file 2 — Supplementary Material 2: Supplementary Fig. 2. Schematic for the experimental outline of using SHH-DAOY and group 3-D283 medulloblastoma cells as extracellular vesicle (EV)-donors and drug-naïve SHH-DAOY cells as EV-recipients to measure the effects of EVs on clonogenic and spheroid growth in 2D and 3D cultures. [file 12964_2025_2241_MOESM2_ESM.tif]

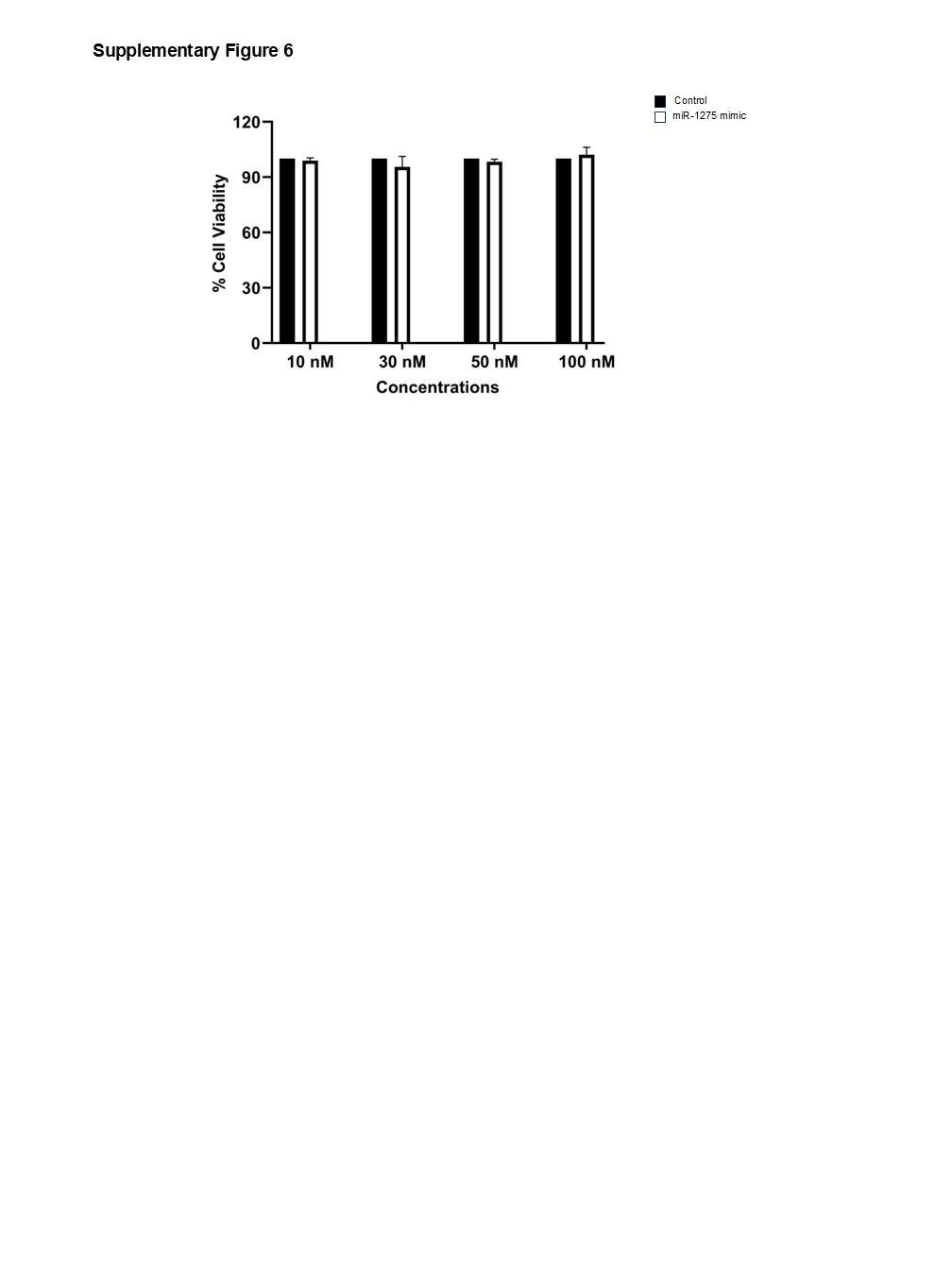

Supplement: Supplementary file 3 — Supplementary Material 3: Supplementary Fig. 3. Representative images of cell colonies and graphs showing the differences in clonogenic growth for recipient SHH-UW228 cells treated with EVs isolated from SHH-DAOY cisplatin-treated and non-treated cells. Recipient SHH-UW228 cells not treated with EVs served as controls. Data represent means ± SEM from three independent experiments, *p < 0.05, **p < 0.01. [file 12964_2025_2241_MOESM3_ESM.tif]

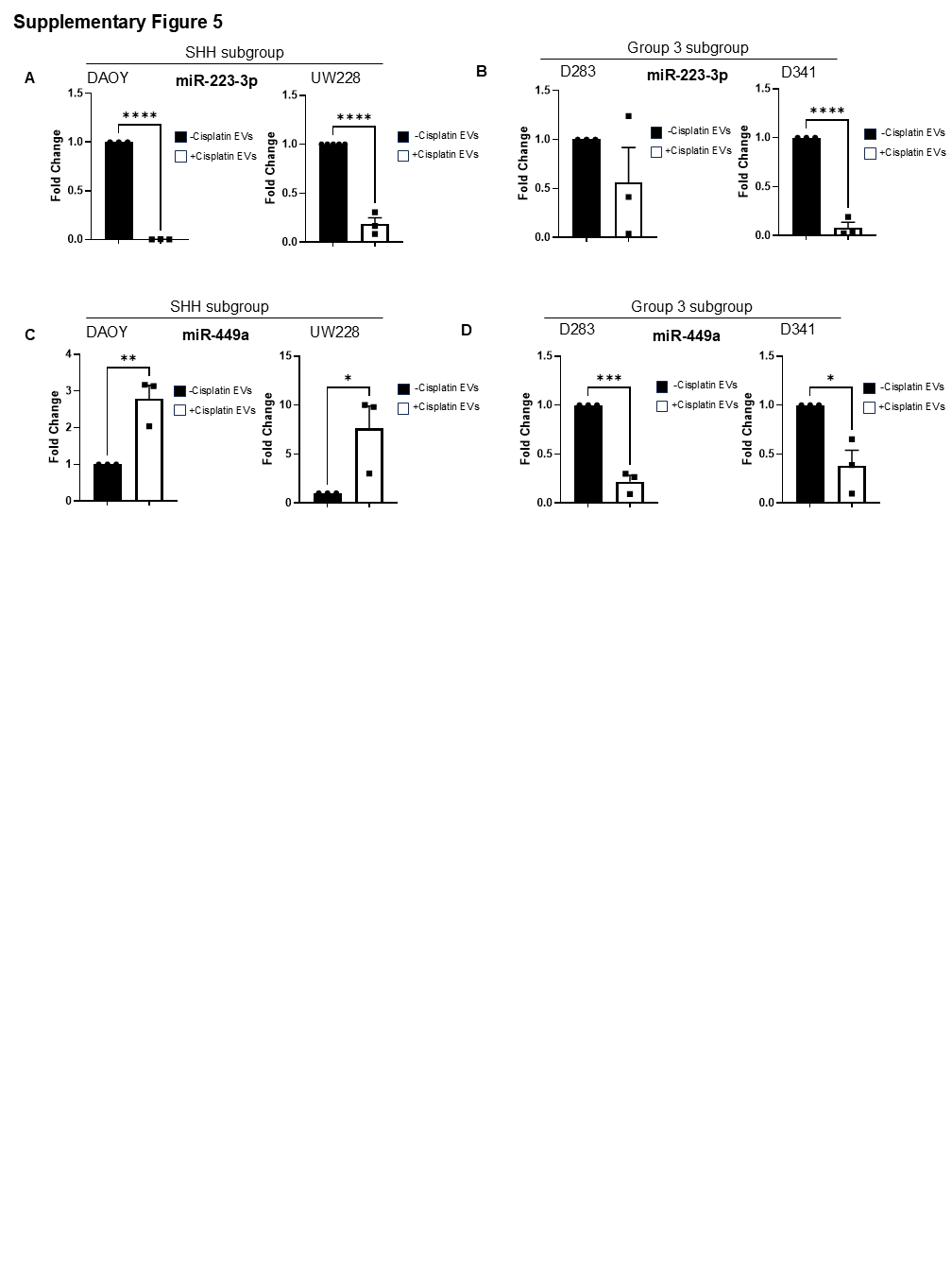

Supplement: Supplementary file 4 — Supplementary Material 4: Supplementary Fig. 4. Western blots showing protein expression of stem cell markers CD133 and ABCG2 after spheroid (medullosphere) formation. GAPDH was used as a protein loading control. [file 12964_2025_2241_MOESM4_ESM.tif]

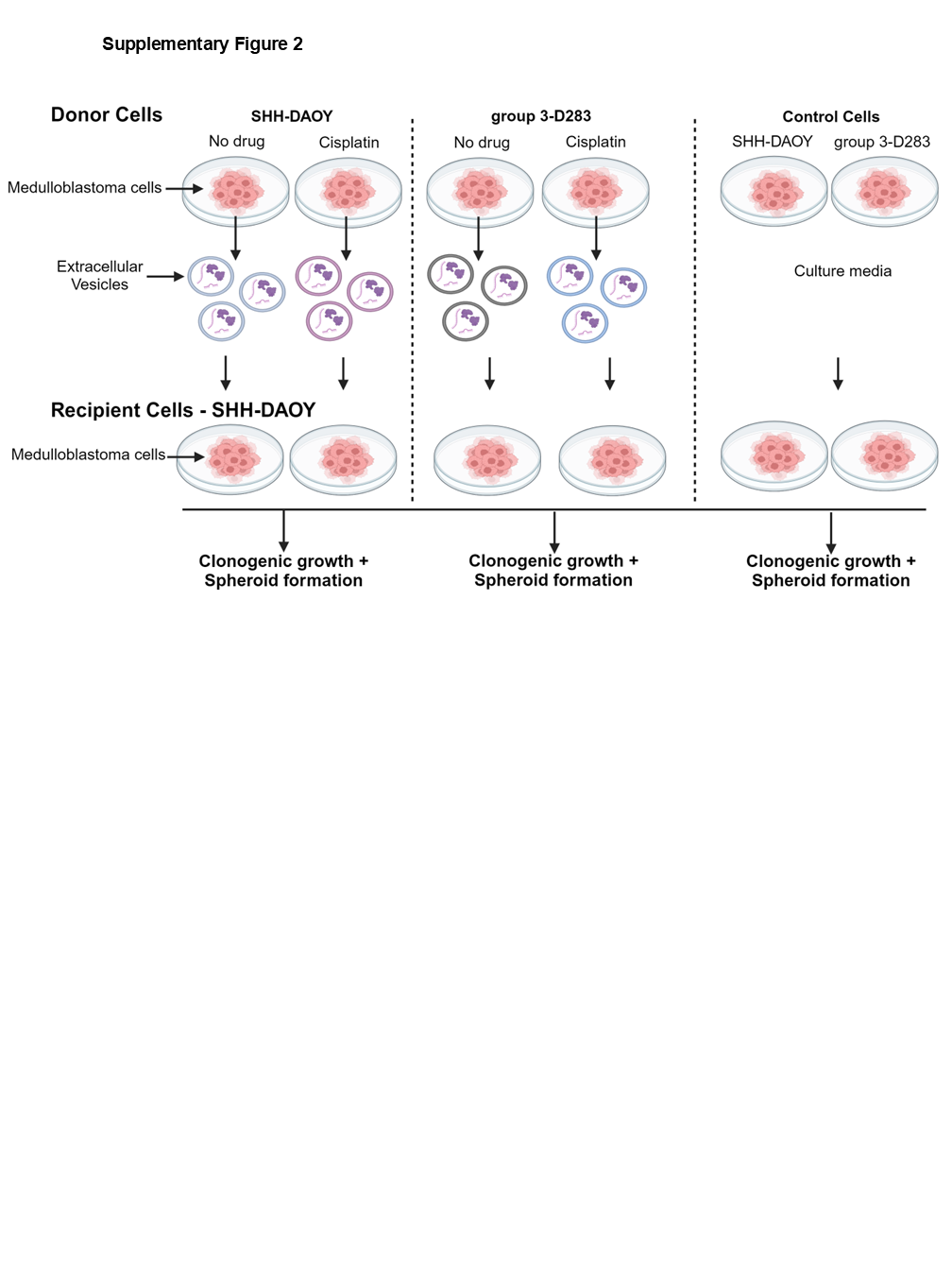

Supplement: Supplementary file 5 — Supplementary Material 5: Supplementary Fig. 5: qPCR validation of candidate miRNAs, (A-B) miR-223-3p and (C-D) miR-449a in extracellular vesicles secreted by SHH-DAOY, SHH-UW228, group 3-D283 and group 3-D341 cells treated with or without cisplatin. U6 served as the reference gene. Extracellular vesicles isolated from non-treated cells served as control. Data represent means ± SEM from three independent experiments, *p < 0.05, **p < 0.01, ***p < 0.001****p < 0.0001. [file 12964_2025_2241_MOESM5_ESM.tif]

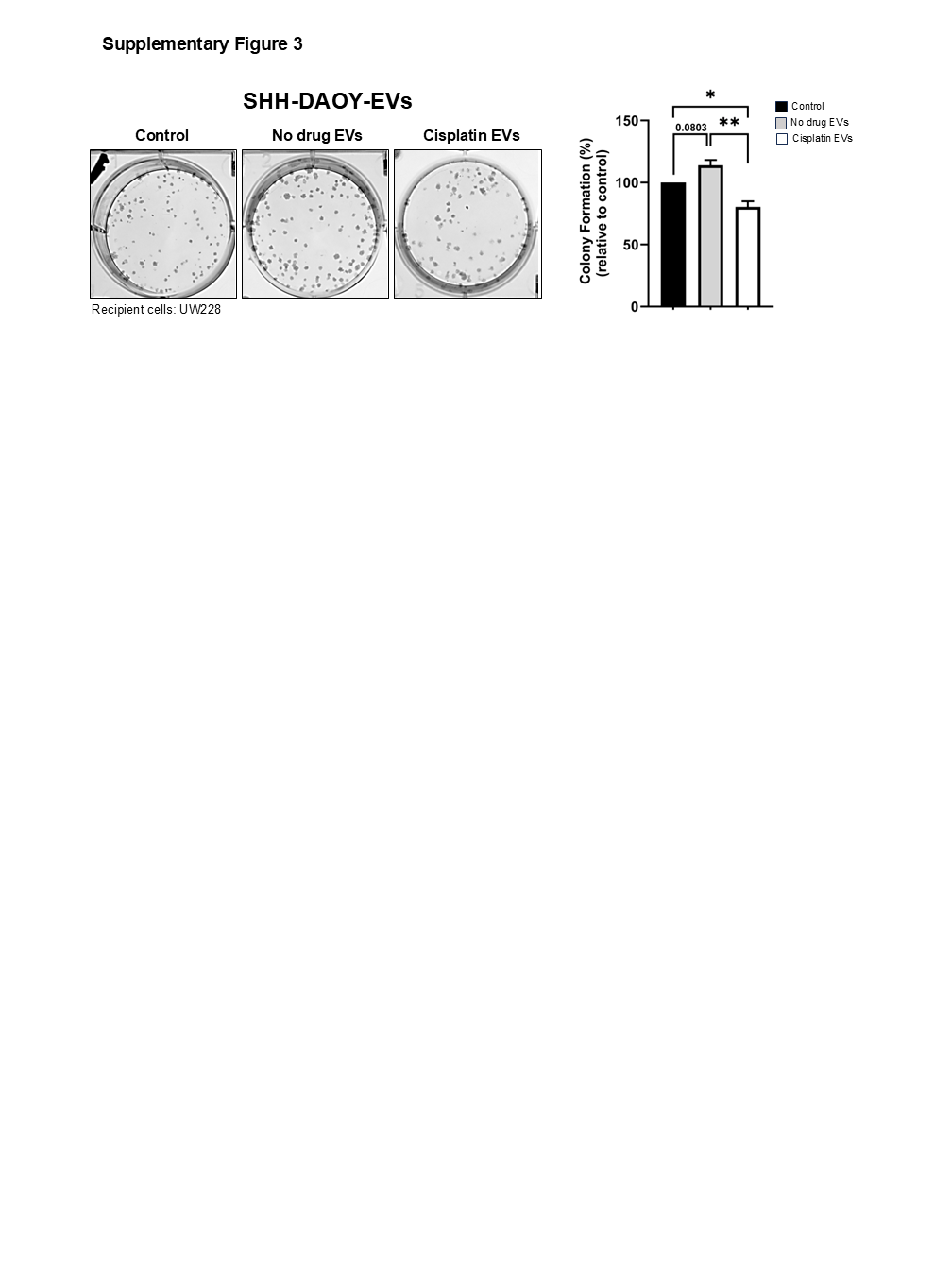

Supplement: Supplementary file 6 — Supplementary Material 6: Supplementary Fig. 6: Cell viability graph of SHH-DAOY cells transfected with increasing concentrations of miR-1275 mimic or non-functional miRNA (control) showing no effect on cell viability. Data represent means ± SEM from three independent experiments. [file 12964_2025_2241_MOESM6_ESM.tif]
